# Peptide linker increased the stability of pneumococcal fusion protein vaccine candidate

**DOI:** 10.3389/fbioe.2023.1108300

**Published:** 2023-01-26

**Authors:** Luciano Zane, Stefanie Kraschowetz, Monalisa Martins Trentini, Vitor dos Santos Alves, Sergio Carneiro Araujo, Cibelly Goulart, Luciana Cezar de Cerqueira Leite, Viviane Maimoni Gonçalves

**Affiliations:** ^1^ Laboratory of Vaccine Development, Butantan Institute, Sao Paulo, Brazil; ^2^ Interunits Graduate Program in Biotechnology, University of Sao Paulo, Sao Paulo, Brazil

**Keywords:** spacer, PspA, pneumolysin, *Streptococcus pneumoniae*, mouse immunization, protease detection, protein structural model, recombinant *Escherichia coli*

## Abstract

*Streptococcus pneumoniae* is a bacterial pathogen exclusive to humans, responsible for respiratory and systemic diseases. Pneumococcal protein vaccines have been proposed as serotype-independent alternatives to currently used conjugated polysaccharide vaccines, which have presented limitations regarding their coverage. Previously in our group, pneumococcal surface protein A (PspA) and detoxified pneumolysin (PdT) were genetically fused and the hybrid protein protected mice against pneumococcal challenge, offered higher cross-protection against different strains and showed greater opsonophagocytosis rate than co-administered proteins. As juxtaposed fusion was unstable to upscale production of the protein, flexible (PspA-FL-PdT) and rigid (PspA-RL-PdT) molecular linkers were inserted between the antigens to increase stability. This work aimed to produce recombinant fusion proteins, evaluate their stability after linker insertion, both *in silico* and experimentally, and enable the production of two antigens in a single process. The two constructs with linkers were cloned into *Escherichia coli* and hybrid proteins were purified using chromatography; purity was evaluated by SDS-PAGE and stability by Western blot and high performance size exclusion chromatography. PspA-FL-PdT showed higher stability at −20°C and 4°C, without additional preservatives. In silico analyses also showed differences regarding stability of the fusion proteins, with molecule without linker presenting disallowed amino acid positions in Ramachandran plot and PspA-FL-PdT showing the best scores, in agreement with experimental results. Mice were immunized with three doses and different amounts of each protein. Both fusion proteins protected all groups of mice against intranasal lethal challenge. The results show the importance of hybrid protein structure on the stability of the products, which is essential for a successful bioprocess development.

## 1 Introduction

*Streptococcus pneumoniae* (pneumococcus) is a bacterial pathogen exclusive to humans, responsible for respiratory and systemic disease (Henrichsen, 1995; Henriques-Normark and Tuomanen, 2013). Pneumonia accounted for 14% of deaths in children under 5 years old globally in 2019, and pneumococcus is the most common cause of bacterial pneumonia (World Health Organization, 2019), being responsible for approximately 50% of pneumonia cases (Roth et al., 2018). Pneumococcal pneumonia affects especially children under 5 years old and the elderly aged 70 and older from lower and middle income countries (Troeger et al., 2017; Roth et al., 2018; Wahl et al., 2018). During the COVID-19 pandemics, the coinfection of pneumococcus and SARS-CoV-2 was related to a weaker antiviral response (Mitsi et al., 2022). The main virulence factor and evasion mechanism of pneumococcus against the human immune system consists of a polysaccharide capsule. Structural and antigenic differences in capsular polysaccharides (PS) have led to the classification of pneumococcal strains into more than 100 serotypes (Geno et al., 2015; Ganaie et al., 2021), which prevalence may vary according to geographic location, age of infected persons (Geno et al., 2015), and symptoms caused during infection (Hausdorff et al., 2000). Although pneumococcal vaccination has been introduced in most countries in the last 3 decades and reduced number of cases and deaths (Rodrigo et al., 2015; Waight et al., 2015; Wahl et al., 2018), currently available PS based vaccines have presented limitations regarding their coverage. The incidence of vaccine serotypes has decreased where vaccines were applied, while serotypes absent in the formulation emerge and become prevalent, leading to serotype replacement and reduced vaccine effectiveness over time (Moore et al., 2015; Waight et al., 2015; Corcoran et al., 2017). The conjugated vaccines are the only vaccines highly effective in children under 2 years of age (Clutterbuck et al., 2012). However, the production of conjugated vaccines consists of several steps resulting in a costly process (Hargreaves et al., 2011; Entwisle et al., 2017), compromising the access of lower income countries, in which pneumococcal diseases mortality rates are the highest (Oligbu et al., 2019). The development of an effective, accessible and serotype-independent pneumococcal vaccine is highly desirable and urgent, considering also the alarming growth in antibiotic resistance among pneumococcal strains (Peyrani et al., 2019).

Conserved protein-based vaccines have been proposed as alternatives to currently available vaccines (Briles et al., 2000a; Miyaji et al., 2013; Gonçalves et al., 2019), which may solve aforementioned issues and still be able to protect risk groups and adult population from pneumococcal diseases. Pneumococcal surface protein A (PspA) is an important virulence factor (Senkovich et al., 2007) and the protein is present in most clinical isolates of *S. pneumoniae* (Ren et al., 2012; Khan and January 2017). Despite its high prevalence, PspA amino acid sequence is variable and presents differences in cross-reactivity (Hollingshead et al., 2000), making it a challenge to select the protein variant capable of inducing cross-reactive antibodies that interact with a large number of different clinically important variants (Moreno et al., 2010). PspA has shown promising preclinical and clinical results (Darrieux et al., 2015; Entwisle et al., 2017); immunization of healthy adults (Nabors et al., 2000) with PspA induced antibodies that passively protected mice in invasive challenges with pneumococcal strains bearing heterologous PspAs (Briles et al., 2000b; 2000a). The PspA variant used in this work is derived from strain St 94/01 (Goulart et al., 2011) and classified as family 1, clade 2. Anti-serum of mice immunized with this protein inhibited the anti-phagocytic activity of PspA and showed high levels of cross-reactivity, promoting opsonization by complement deposition of pneumococcal strains expressing heterologous PspA, indicating strong cross-protection.

Another important pneumococcal protein vaccine candidate is pneumolysin (Ply), a cholesterol-dependent toxin involved in several evasion mechanisms, such as hemolysis, complement system activation and apoptosis induction (Braun et al., 2002; Marriott et al., 2008). Ply is highly conserved among pneumococcal strains (Mcchlery et al., 2004; Jefferies et al., 2007; Han and Zhang, 2019), and has already been evaluated as a vaccine in several studies, whether used alone, in fusion or co-administered with other proteins and the results were promising (Ogunniyi et al., 2007; Goulart et al., 2013; Kamtchoua et al., 2013; Leroux-Roels et al., 2014; Prymula et al., 2014). Ply needs to be detoxified (generating a pneumolysoid) to be used in a vaccine. The pneumolysoid used in this work was PdT, genetically detoxified pneumolysoid with three point mutations: Cys428→Gly, Trp433→Phe, and Asp385→Asn, which presents only 0.0001% of the wild-type toxin cytotoxicity and no complement activation (Berry et al., 1995). We have previously constructed a recombinant hybrid molecule using PspA 94/01 and PdT (Goulart et al., 2013). Comparing the fusion protein to each protein alone or co-administered, rPspA-PdT hybrid protein induced increased cross-reactive antibodies in mice and enhanced complement deposition onto heterologous strains, in addition to protecting mice against lethal challenge with pneumococcal strains bearing heterologous PspAs. Albeit showing promising results, stability issues with rPspA-PdT during purification and storage were verified while carrying out scaling up experiments.

Molecular linkers have presented several advantages in recombinant fusion proteins, such as improving stability, enhancing biological activity, increasing expression yield and altering the pharmacokinetic profiles of proteins (Chen et al., 2013; Arai, 2021). In order to overcome the stability problems of the rPspA-PdT hybrid, linkers were inserted between the proteins, resulting in different conformations of the hybrid molecule: rPspA-RL-PdT, with the proteins joined by a rigid helix-forming linker (Arai et al., 2001), and rPspA-FL-PdT, joining the proteins by a flexible linker composed of glycine and serine (Maeda et al., 1996; Maeda et al., 1997). These linkers were chosen to promote the separation of the protein domains by the stiff α-helical structure, ensuring a distance between them (Arai 2021), or to allow more conformational freedom to the hybrid molecule because of the glycine and serine residues, not interfering with main protein domains nor hindering their folding (Ceballos-Alcantarilla and Merkx, 2021). Another type of linker described in the literature is composed of proline and alanine (Zhao et al., 2008), but the C-terminal portion of PspA sequence is composed of these amino acids (called proline-rich region or PRR), representing a “natural” linker between PspA and PdT in rPspA-PdT molecule. Zhao et al. (2008) achieved increased stability of a fusion protein using these linkers, by avoiding aggregate formation and decreasing hydrolysis susceptibility, and also enhanced antiviral activity of their protein. In this study, the rigid and flexible molecular linkers are used to evaluate if they are able to increase PspA-PdT stability, using both *in silico* predictions and experimental analyses, and still protect mice against lethal pneumococcal challenge.

## 2 Materials and methods

### 2.1 Scaling up culture and purification trials

*Escherichia coli* M15 strain transformed with the plasmid pQE-30 containing the pspa94-pdt fusion gene from Goulart et al. (2013) was cultured in 10 L bioreactor at 30°C using 6 L chemically defined HDF medium (Seeger et al., 1995), kanamycin (25 μg/mL) and ampicillin (100 μg/mL). Induction was carried out using 1 mM isopropyl ß-D-1-thiogalactopyranoside (IPTG). Cells were harvested by centrifugation and frozen at −80°C prior to use. Before purification, cells were suspended in lysis buffer (10 mM Na-phosphate, 0.1% Triton X-100, 5 mM EDTA, 5 mM PMSF), lysed with a high pressure continuous homogenizer PandaPLUS 2000 (GEA Group, Düsseldorf, Germany), incubated with 0.1% cetyltrimethylammonium bromide (CTAB) agitating for 1 h to precipitate endotoxins (Figueiredo et al., 2017). The lysate was centrifuged and the soluble fraction was filtered. Several purification trials were carried out using anion exchange (Q-Sepharose FF), immobilized metal affinity (IMAC-Sepharose FF), hydrophobic interaction (Phenyl-Sepharose FF High Sub), and size exclusion chromatographic (Sephacryl S-200 HR) methods.

### 2.2 Western blot

Western blot using anti-pneumococcal whole cell vaccine (PWCV) polyclonal serum (Gonçalves et al., 2014), anti-PspA94 and anti-PdT (Goulart et al., 2013) was performed with rPspA-PdT samples before (clarified lysate) and after purification. These samples were applied three times in the SDS-PAGE (10%) and transferred to the membrane, which was stained with Ponceau S (Supplementary Figure S1) and split into three parts, each one incubated with one of the antibodies. Detection was achieved with 3,3′-diaminobenzidine (DAB).

### 2.3 Casein digestion assay

Proteolytic activity on casein was determined using Gonçalves et al. (2003) protocol. Casein (1% w/v) was used as a substrate in 0.02 M Tris-HCl buffer, pH 8.0, 0.02% sodium azide. Casein solution (0.4 mL) was incubated with 0.1 mL of sample at 37°C for 48 h. Non-hydrolyzed casein was precipitated by adding 0.4 mL of 50% (w/v) trichloroacetic acid (final concentration: 10%), tubes were kept on ice for 30 min and centrifuged at 17,000 g for 10 min; absorbance at 280 nm was measured before and after incubation. Trypsin (Sigma-Aldrich, San Louis, MO, United States) at 0.5 mg/mL and distilled water were used as positive and negative controls, respectively.

### 2.4 Zymographic assay

The presence of hydrolytic enzymes in the samples was also evaluated by in-gel zymography (Fernández-Resa et al., 1995; Leber and Balkwill 1997; Vandooren et al., 2013). Sodium dodecyl sulfate (SDS) polyacrylamide gels 12% were copolymerized with either 0.1% gelatin or 0.1% casein. Samples were prepared under non-reducing conditions, trypsin 0.5 mg/mL solution was used as positive control and electrophoresis was carried out for 90 min at 120 V. After the run, gels were washed twice for 30 min and twice for 10 min with 2.5% Triton X-100 solution for SDS removal, and then incubated in collagenase buffer (50 mM Tris-HCl, 200 mM NaCl, 5 mM CaCl_2_, pH 7.6) for 18 h at 37°C. Gels were stained (25% ethanol, 5% acetic acid, 0.1% Coomassie Blue G-250) overnight and destained (10% ethanol, 5% acetic acid) for 2 h. Control gels were cast without substrates to show protein bands of the samples (Supplementary Figure S2).

### 2.5 Assays for detection of serine- and metalloproteases

For the detection of serine proteases, the chromogenic reagent Nα-benzoyl-L-arginine-p-nitroanilide, L-BAPNA (Hieng et al., 2004), was used as substrate, as described by Knittel et al. (2016). This substrate was prepared at a concentration of 115 mM in 50 mM Tris-HCl buffer, pH 7.5. Incubation of substrate and sample was carried out in 96-well plates containing: 160 μl of 50 mM Tris-HCl buffer pH 7.5, 20 μl of substrate solution and 20 μl of sample at different concentrations (diluted in the same buffer) for 40 min at 37°C. *Bothrops jararaca* crude venom (5 μg per well) and buffer instead of sample were used for the positive and negative control, respectively. After incubation, absorbance was measured at 405 nm.

For the detection of metalloproteases, Abz-AGLA-EDDnp reagent (Freitas-de-Sousa et al., 2017; Mancuso et al., 2018) was used as a substrate at a final concentration of 200 μM, according to Knittel et al. (2016). This substrate was diluted before use to 400 μM in 50 mM Tris-HCl buffer with 10 mM CaCl_2_, 150 mM NaCl and 0.05% (v/v) Brij^®^ 35, pH 7.5. The incubation of the substrate with the sample was carried out in 96-well plates containing 50 μl of diluted substrate and 50 μl of sample at different concentrations. Absorbance was monitored for 10 min, with measurements every 1 min at 320 and 420 nm wavelengths. Jararhagin isolated from *B. jararaca* venom (5 μg/mL) and buffer instead of sample were used for positive and negative control, respectively. Specific activity of metalloproteinases was calculated in relative fluorescence units (RFU/min/µg).

### 2.6 N-terminal sequencing

For determination of the degradation site of the hybrid molecule, a sample of degraded rPspA94-PdT was loaded into immobilized metal affinity chromatography (IMAC) to separate both parts of the degraded fusion. The N-terminal His-tagged fragment remained bound to the resin, while the C-terminal fragments (without His-tag) were recovered in the flowthrough. Then, the flowthrough fraction was loaded into high performance liquid chromatography (HPLC) on a butyl (C4) stationary phase column. For the equilibration, 0.1% trifluoroacetic acid (TFA) solution was used and elution was carried out with the same solution and gradient from 0% to 100% acetonitrile. Elution absorbance at 280 nm was monitored in real time on a chromatogram and the major peak was manually collected. This main peak was concentrated and its N-terminus portion was sequenced using Edman degradation method (Edman et al., 1950).

### 2.7 New gene constructs

Two new constructs were proposed, which would join the antigens with different molecular linkers: A flexible linker (FL), formed by glycines and serines, whose sequence is N- GGGGSGGGGS- C; and a rigid linker (RL), which forms an alpha-helix structure, whose sequence is N- AEAAAKEAAAKA -C. In addition, within the proline-rich region, the non-proline sequence block (NonPro), and the last block of prolines of PspA were removed from this new construct (Hollingshead et al., 2000), since recombinant PspA molecules that do not have this non-Pro region, such as PspA4Pro (Moreno et al., 2010; Figueiredo et al., 2017) and PspA3 (Carvalho et al., 2012), were more stable than recombinant PspA245 in whose sequence the region was maintained (Barazzone et al., 2011). Also, the new constructs were cloned into pET28a, eliminating nine pQE-30 derived amino acids from beginning of the tandem fusion protein, three (RGS) before six histidine tag (His-tag), and another six (GSACEL) after His-tag and before the first PspA amino acid.

Overlap extension PCR was carried out with high-fidelity polymerase (Q5 high fidelity DNA polymerase; New English Biolabs, Ipswich, MA, United States), where two PCR are performed in parallel to amplify the genes of each protein with a linker sequence and a third PCR in series to join the products of the previous reactions (Ho et al., 1989; Reikofski and Tao 1992; Bryksin and Matsumura 2010). The plasmid pQE-30 carrying *pspa94-pdt* gene (Goulart et al., 2013) was used as template to construct the two new hybrid proteins. Primers were designed to insert the linker region between *pspa* and *pdt* (Table 1). Optimal conditions for the reactions were defined after evaluation of different annealing temperatures (70°C–55°C) and different concentrations of magnesium chloride (1–4 mM). The genes were amplified in a thermocycler at the following optimal conditions: 2 min at 94°C, 30 cycles of 94°C for 30 s, 60°C for 60 s and 72°C for 90 s each and 72°C for 5 min, using 4 mM MgCl_2_. PCR products were analyzed using agarose gel electrophoresis. Gel bands were purified using a GFX PCR DNA Purification and Gel Band Purification Kit (Cytiva, Marlborough, MA, United States) and nucleic acid concentration was determined using NanoDrop (Epoch Microplate Spectrophotometer, Bio-Tek).

**TABLE 1:** Primers used in overlap PCR to build the new fusion proteins with molecular linkers inserted between the antigens. Forward and reverse primers are indicated as F or R, respectively.

rPspA-FL-PdT	1st PCR	*pspa* + flexible linker sequence	**A:** 5′ CCA​TGG​CAG​AAG​CGC​CCG​TAG​CTA 3’—F
			**B:** 5′ TGA​ACC​TCC​GCC​CCC​AGA​CCC​GCC​TCC​ACC​TGG​A GCTGGAGCTG 3′—R
		flexible linker sequence + *pdt*	**C:** 5′ GGT​GGA​GGC​GGG​TCT​GGG​GGC​GGA​GGT​TCA​ATG​GCA​A ATAAAGCAGTAAATG ′—F
			**D:** 5′ CTC​GAG​GTC​ATT​TTC​TAC​CTT​ATC​CTC​TAC​CTG​A 3′—R
	2nd PCR	Amplification of hybrid gene with flexible linker	**A:** 5′ CCA​TGG​CAG​AAG​CGC​CCG​TAG​CTA 3′—F
			**D:** 5′ CTC​GAG​GTC​ATT​TTC​TAC​CTT​ATC​CTC​TAC​CTG​A 3′—R
rPspA-RL-PdT	1st PCR	*pspa* + rigid linker sequence	**A:** 5′ CCA​TGG​CAG​AAG​CGC​CCG​TAG​CTA 3′—F
			**B:** 5′ TTC​TTT​AGC​TGC​AGC​TTC​TTT​AGC​TGC​AGC​TTC​TGC TGGAGCTGGAGCTGG 3′—R
		rigid linker sequence + *pdt*	**C:** 5′ GCT​AAA​GAA​GCT​GCA​GCT​AAA​GAA​GCT​GCA​GCT​A AAG​CTA​TGG​CAA​ATA​AAG​CAG​TAA​ATG​ACT​TTA​TAC​TAG​C 3′—F
			**D:** 5′ CTC​GAG​GTC​ATT​TTC​TAC​CTT​ATC​CTC​TAC​CTG​A 3′—R
	2nd PCR	Amplification of hybrid gene with rigid linker	**A:** 5′ CCA​TGG​CAG​AAG​CGC​CCG​TAG​CTA 3′—F
			**D:** 5′ CTC​GAG​GTC​ATT​TTC​TAC​CTT​ATC​CTC​TAC​CTG​A 3′—R

### 2.8 Cloning and plasmid constructs

High-fidelity polymerase used for the PCR produces fragments with blunt ends, so the PCR products were purified and an adenylation reaction with Taq DNA polymerase (GoTaq DNA polymerase, Promega) was carried out before inserting the PCR product into the cloning vector. Reaction was carried out with a final concentration of 2.5 mM MgCl_2_ and 0.2 mM dATP in a thermocycler for 18 min at 70°C followed by 2 min at 72°C. Reaction product was used directly in the ligation reaction with the cloning vector pGEM-T Easy Vector (Promega, Madison, WI, United States). Competent *E. coli* DH5α cells were transformed with the plasmids carrying the genes of *pspa-fl-pdt* (flexible) or *pspa-rl-pdt* (rigid). With the constructs obtained, colonies were screened; restriction maps and sequencing were performed. The gene sequences were submitted to GenBank database under the accession numbers OP871266 (*pspa-fl-pdt*) and OP871267 (*pspa-rl-pdt*). Minipreparation was performed with Illustra plasmidPrep Mini Spin kit (Cytiva, Marlborough, MA, United States). Commercial plasmid pET-28a (Novagen) was digested with the enzymes *Nco*I and *Xho*I and treated with calf intestinal alkaline phosphatase (Promega, Madison, WI, United States) to remove the phosphate groups from the 5′ends and prevent their recircularization. Subsequently, cloning vectors with the inserts were digested with the same restriction enzymes and inserts were cloned into the expression vector pET-28a, providing a C-terminal His-tag to both fusion proteins. Resulting constructs pET-28a-*pspa-rl-pdt* (rigid) or pET-28a-*pspa-fl-pdt* (flexible) were respectively transformed into the expression strains *E. coli* BL21 (DE3) (Novagen) and *E. coli* BL21 (DE3) Rosetta (Novagen).

### 2.9 *E. coli* cultivation for protein production

Glycerol stocks (10%) in chemically defined HDF medium (Korz et al., 1995) were prepared for each producer clone, ensuring the cells were still in exponential growth phase. Kanamycin (30 μg/mL) was added for both clones and chloramphenicol (34 μg/mL) only for *E. coli* BL21 (DE3) Rosetta. Main cultures were performed at 30°C in Tunair™ shaken flasks using HDF medium with glycerol as carbon source, and protein production was induced in mid-log-phase cultures with 0.4 mM IPTG. Five hours after induction, cells were harvested by centrifugation and frozen at −20°C.

### 2.10 Hybrid protein purification

Cell pellets were suspended with lysis buffer (20 mM Na-phosphate, 500 mM NaCl, 0.1%, Triton X-100, 1 mM PMSF), lysed at high pressure continuous homogenizer PandaPLUS 2000 (GEA Group, Düsseldorf, Germany) and incubated with 0.1% CTAB agitating for 1 h to precipitate endotoxins (Figueiredo et al., 2017). Both rPspA-FL-PdT and rPspA-RL-PdT were produced in soluble form, thus, soluble fraction was collected after centrifugation, pH was adjusted to 7.5 and imidazole was added to a final concentration of 1 mM. The material was then filtered (0.45 µm) prior to chromatography. Hybrid proteins were purified through IMAC with Ni^2+^ charged MiniChrom Fractogel^®^ Metal Chelate (Merck Millipore, Darmstadt, Germany) column in an ÄKTA avant system. Elution was carried out with 20 mM Na-phosphate pH 7.5, 500 mM NaCl, 200 mM imidazole, and elution fraction was loaded into Sephacryl S-200 (Cytiva, Marlborough, MA, United States) size exclusion column. Elution was then carried out with Na-phosphate buffer saline pH 7.4. Purified fractions were sterile filtrated, stored at −20°C, and used within 3 months for the immunizations. Total protein was quantified by DC™ Protein Assay Reagent Kit (Bio-Rad, Hercules, CA, United States) and bovine serum albumin (BSA) was used as standard. Purity was assessed by sodium dodecyl sulfate-polyacrylamide gel electrophoresis (SDS-PAGE) and gel densitometry (Loccus Pix DS 5000 and LabImage 1D; Loccus, Cotia, Brazil). The relative quantity of target proteins was calculated by Eq. 1

$$relative\;quantity\;\left(\%\right)=\frac{intensity\;of\;target\;protein\;band}{\sum intensity\;of\;all\;bands\;in\;the\;lane\;}\times 100$$ (1)

### 2.11 Stability test

Purified samples obtained from size exclusion chromatography were used to evaluate the stability of rPspA-FL-PdT and rPspA-RL-PdT periodically at 4°C and −20°C. Aliquots were prepared for each period of time (1, 2, 3, 6, and 12 months) and evaluated by SDS-PAGE and Western blot. Chemiluminescence detection was performed with anti-polyHistidine-Peroxidase conjugated antibody (Sigma-Aldrich, San Louis, MO, United States). Additionally, high performance size exclusion chromatography (TSKgel G3000PWXL column, Tosoh Bioscience, Tokyo, Japan) was carried out to quantitatively assess the stability of the fusion proteins, using 18-month sterile filtrated purified samples stored in Na-phosphate buffer saline (pH 7.4) at 4°C or −20°C. The fusion protein peak and all other peaks of the chromatogram detected by UV_280_ were analyzed and compared regarding percentage of total chromatogram area (%area).

### 2.12 Secondary structures of purified hybrid proteins

Secondary structures and stability of purified rPspA-FL-PdT and rPspA-RL-PdT at different temperatures were verified by circular dichroism (CD) spectroscopy, using a JASCO J-810 spectropolarimeter (Japan Spectroscopic, Tokyo, Japan). rPspA-FL-PdT and rPspA-RL-PdT samples were prepared in 20 mM phosphate buffer pH 7.0. Measurements were taken from 185 to 260 nm and final CD spectra were obtained from the mean of five measurements. The fusion proteins were heated (1°C/min) from 2°C to 98°C and cooled back to 2°C (1°C/min), and their melting temperature (Tm) was calculated using Origin software (OriginLab Corporation) by fitting the sigmoid curves using Boltzmann function and finding the minimum value of its derivative at *X*-axis, which represents the sigmoid inflection point. Deconvolution was calculated based on the Dichroweb (Whitmore and Wallace, 2004) online database and the CDSSTR (Sreerama and Woody, 2000) algorithm.

### 2.13 Tertiary structure prediction and validation of hybrid proteins

The user-friendly Colab version of AlphaFold2 software (https://colab.research.google.com/github/sokrypton/ColabFold/blob/main/AlphaFold2.ipynb) was used to predict the molecular structure of the hybrid proteins, with and without linkers, using default MMseqs2 parameters (Mirdita et al., 2022). The predicted unrelaxed models presented as “rank 1” by AlphaFold2 were used as input in GalaxyWEB (Heo et al., 2013) for refinement (https://galaxy.seoklab.org/cgi-bin/submit.cgi?type=REFINE) and BIOVIA Discovery Studio Visualizer (Dassault Systèmes, Vélizy-Villacoublay, France) was used to visualize the refined models. GalaxyWEB output generates five new models and provides information regarding molecular dynamics; the chosen model should present the highest “GDT-HA” (global distance test - high accuracy), “RMSD” (root-mean-square deviation), “Rama favored”, and the least “MolProbity”. The refined model for each protein was used as input in PROCHECK (Laskowski et al., 1993) (https://saves.mbi.ucla.edu/) for stereochemical evaluation by Ramachandran plot and in ProSA-web (Sippl, 1993; Wiederstein and Sippl, 2007) (https://prosa.services.came.sbg.ac.at/prosa.php) for comparing Z-scores with solved protein structures. Additionally, Qualitative Model Energy Analysis (Benkert et al., 2009) (QMEAN, https://swissmodel.expasy.org/qmean/) was used for model quality estimation of each protein and for identification of potentially problematic regions in the sequences.

### 2.14 Pneumococcal strain

Pneumococcal strain A66.1 (serotype 3 carrying PspA from family 1 and clade 2) was maintained as frozen stock (−80°C) in Todd-Hewitt broth supplemented with 0.5% yeast extract (THY) with 10% glycerol. Before the experiments, the isolate was plated on blood agar for overnight growth, then cultured in THY up to OD_600nm_ 0.4–0.5 and harvested by centrifugation. Newly prepared stocks were frozen with 10% glycerol and serial dilutions were plated on blood agar for determining viable cell concentration by colony-forming units (CFU/mL).

### 2.15 Mouse immunization and pneumococcal challenge

This study was carried out in accordance with the rules issued by the National Council for Control of Animal Experimentation (CONCEA). The protocol was approved by the Ethic Committee on Animal Use of the Butantan Institute (CEUAIB), Permit Number: 1905090218. Six-week-old female specific-pathogen-free (SPF) BALB/c mice (5-6 animals per group), from the Medical School of the University of São Paulo (São Paulo, Brazil), were immunized subcutaneously (total volume: 200 µl) with three doses of rPspA-FL-PdT or rPspA-RL-PdT, varying amounts of protein per dose (5, 10 or 20 µg) at 15-day intervals, using sterile saline solution 0.9% and aluminum hydroxide (Alum) as adjuvant (50 µg per dose), totalizing seven groups. On the day of the immunization, frozen aliquots stored at −20°C from recently purified proteins were thawed and adsorbed with Alum before inoculating them to the animals. The same purification batch of each protein was used for the whole experiment. The adjuvant alone in saline was used as negative control. Fourteen days after each immunization, the animals were bled by retro-orbital puncture and antibody production was evaluated by enzyme-linked immunosorbent assay (ELISA). 21 days after the third immunization, mice were anesthetized through the intraperitoneal route with a xylazine/ketamine solution (25 mg/Kg of xylazine and 50 mg/Kg of ketamine) and challenged through the intranasal route with 1 × 10^5^ CFU of strain A66.1. The suspension of pneumococci in 50 μl of sterile saline solution 0.9% was inoculated into one nostril. This procedure ensures the solution reaches the lungs and does not remain only in the upper airways (Rodrigues et al., 2018). After challenge, mice were monitored twice per day, and lethargic animals with reduced mobility were euthanized with a lethal dose of a xylazine/ketamine solution (60 mg/Kg of xylazine and 300 mg/Kg of ketamine). Survival rate curves were analyzed by Log-rank test (Mantel-Cox).

### 2.16 Measurement of antibodies by ELISA

ELISA was carried out in 96-well plates (Nunc MaxiSorp^®^, Thermo Fisher Scientific) coated overnight at 4°C with 5 μg rPspA-FL-PdT or rPspA-RL-PdT in 0.1 M carbonate-bicarbonate buffer pH 9.6 (depending on the group analyzed). Non-coated wells were used as blocking control. Plates were incubated for 30 min at 37°C, washed three times with PBS-Tween 0.05% (PBST) and then blocked with 200 μl of 5% skim milk in PBS for 1 h at 37°C. The plates were washed three times with PBST. In the first assay (Supplementary Figure S3), pooled samples of each dose of each group were prepared, diluted in PBS with 1% BSA, with an initial dilution factor of 1:1,000 for sera after the first dose and 1:4,000 after the second and third doses. Then, 200 μl were added to the first wells and two-fold serial dilutions were carried out to the end of the plate so that every well contained 100 μl. In the second assay, a previous dilution was chosen based on titration of the pools, and the sera from all animals were individually diluted in PBS with 1% BSA and added to the plate. Animal sera were not added to the wells corresponding to the blank (PBS with 1% BSA). Plates were incubated for 2 h at 37°C. Plates were washed three times with PBST and then 100 μl of horseradish peroxidase-conjugated anti-mouse IgG (Sigma-Aldrich, San Louis, MO, United States) diluted in PBS with 1% BSA (1:5,000) were added, and then plates were incubated for 1 h at 37°C. Plates were washed three times with PBST and the development was performed with a solution of OPD (1.0 mg/mL) and H_2_O_2_ (5 μL/mL of 30% H_2_O_2_) in citrate-phosphate buffer pH 5.0 (0.1 M sodium citrate; 0.2 M monobasic sodium phosphate) for 15 min at room temperature in the dark. Reaction was stopped by adding 50 μl of 4 M H_2_SO_4_ and absorbance was measured at 492 nm wavelength. The same protocol was followed to analyze antibodies against individual proteins, but the coating was carried out with rPspA94 or rPdT and all groups were analyzed for each protein. In these experiments, a titration of pooled groups (Supplementary Figure S4) was also carried out first and then, each animal was analyzed individually. Statistical analysis of the specific IgG produced in each group was performed by one-way ANOVA with a Tukey’s Multiple Comparison Test.

## 3 Results

### 3.1 Degradation of rPspA-PdT after purification

**FIGURE 1:**
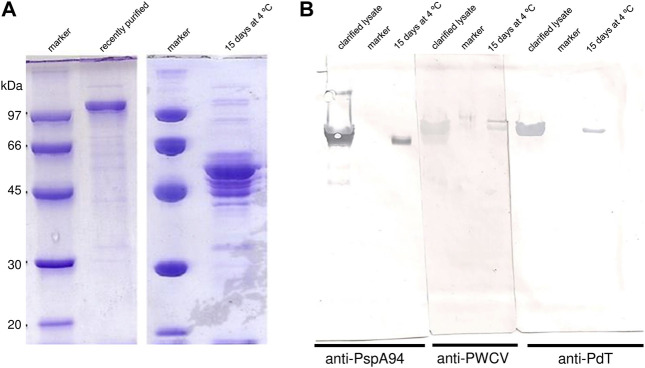
Degradation of PspA-PdT observed during purification trial. **(A)** SDS-PAGE 10% assessing PspA-PdT purity and stability. PspA-PdT sample after purification by hydrophobic interaction, 0 and 15 days after storage at 4°C were loaded. **(B)** Western blot assessing PspA-PdT stability using different antibodies for detection; clarified lysate, marker and 15-day purified PspA-PdT stored at 4°C were loaded in triplicates, transferred, the membrane was split in three parts and incubated with different antibodies: anti-PspA94, anti-PWCV (pneumococcal whole cell vaccine) and anti-PdT. Detection was achieved with 3,3′-diaminobenzidine (DAB). The membrane incubated with anti-PWCV shows PdT (above) and PspA (below), while the other parts show them individually, and no detection was observed in the corresponding size of the fusion protein.

In the first purification trial, rPspA-PdT purity achieved 85.5% after purification (Figure 1A). Fifteen days after the purification process, the sample was analyzed by SDS-PAGE and degradation of the hybrid protein was observed (Figure 1A). Thus, Western blotting was carried out using anti-PspA94, anti-PWCV polyclonal serum or anti-PdT to detect both antigens in the clarified lysate and in the purified sample where degradation was observed (Figure 1B). When the membrane was incubated with anti-PWCV, two bands at the expected molecular weights of the individual rPspA94 and rPdT proteins were observed (Figure 1B), which may indicate that the hybrid protein was breaking between the two proteins genetically linked in this construction. The lanes incubated with anti-PspA94 or anti-PdT indicated the lower band corresponds to PspA moiety and the upper band to PdT (Figure 1B). Clarified lysate lanes also showed degradation of the fusion protein, but the individual antigens were not clearly separated as on purified sample. From these results, the hypothesis that the protein was degrading during the purification process started to be investigated.

It is also important to point out that rPspA presents “apparent molecular mass” in SDS-PAGE different than expected (Yother et al., 1992), which explains why electrophoretic profile of rPspA-PdT hybrid molecules differs from their theoretically molecular mass of ∼88 kDa.

### 3.2 Casein digestion and zymographic assays

**FIGURE 2:**
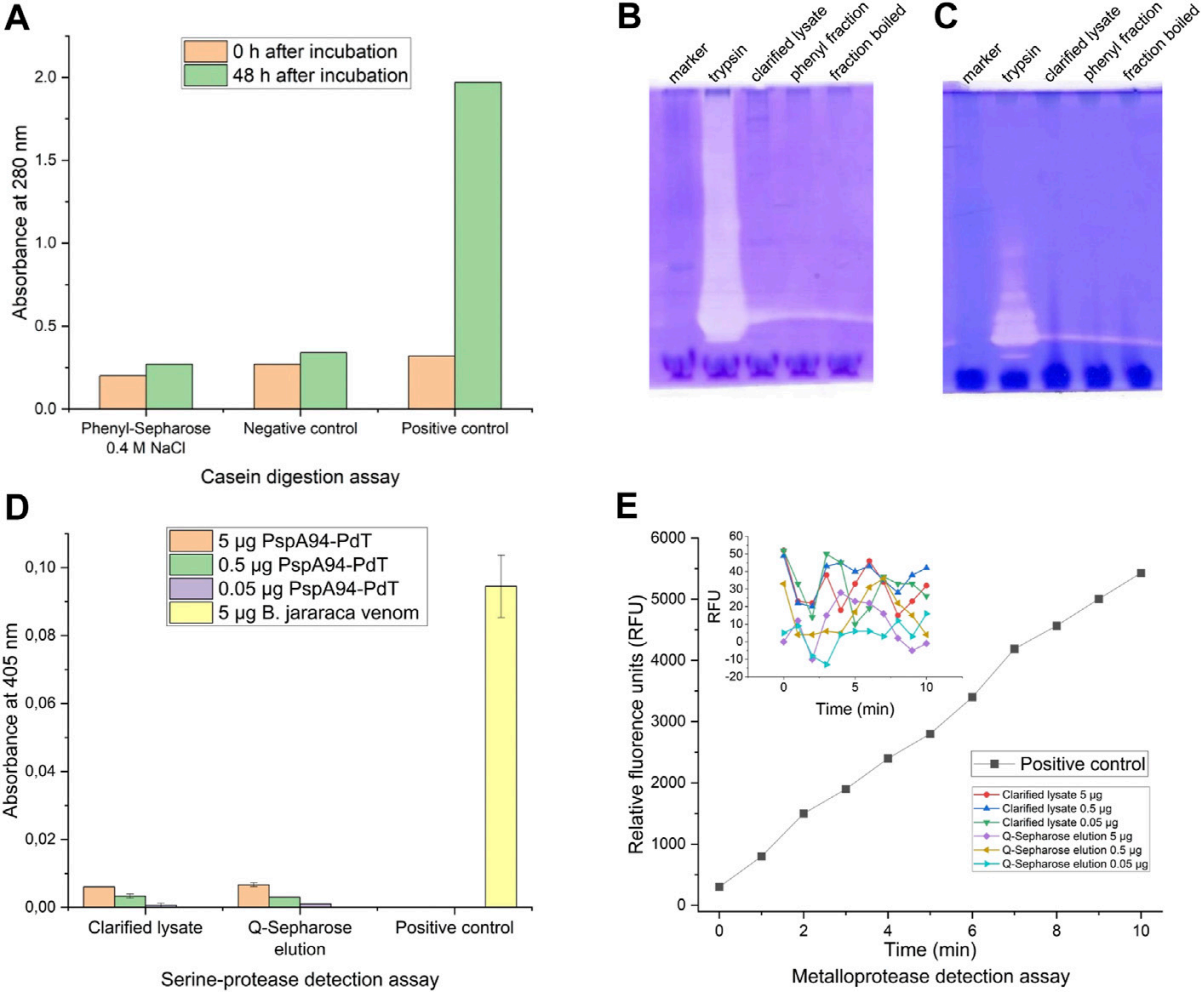
Protease activity detection assays. **(A)** Casein digestion assay for detection of proteolytic activity using partially purified sample from a Phenyl-Sepharose elution fraction (0.4 M NaCl), where degradation was observed 15 days after storage at 4°C; trypsin (0.5 mg/mL) and distilled water were used as positive and negative controls, respectively. **(B)** In-gel zymography with 0.1% gelatin as substrate; lanes: molecular weight marker; trypsin as positive control; recently clarified lysate; partially purified sample from a Phenyl-Sepharose elution fraction where possible degradation was observed; the same Phenyl-Sepharose fraction boiled for 10 min before load. **(C)** Same as B but with 0.1% casein as substrate; **(D)** Detection of serineprotease activity after incubation with chromogenic substrate L-BAPNA in different dilutions of recently clarified lysate and partially purified fraction from Q-Sepharose (0.2 M NaCl), where degradation was observed; *B. jararaca* venom was used as positive control. **(E)** Detection of metalloprotease activity after incubation with chromogenic substrate Abz-AGLA-EDDnp in different dilutions of recently clarified lysate and partially purified fraction from Q-Sepharose (0.2 M NaCl), where degradation was observed; isolated jararhagin from *B. jararaca* venom was used as positive control.

The presence of proteolytic activity was investigated by two methods in recently clarified lysate and/or a partially purified sample from a Phenyl-Sepharose chromatography (containing 20% ethanol). Casein digestion assay was carried out using partially purified sample from a Phenyl-Sepharose elution fraction containing 0.4 M NaCl, where degradation was observed 15 days after storage at 4°C. No proteolytic activity was observed in this sample (Figure 2A). The presence of hydrolytic enzymes in the samples was also evaluated by in-gel zymography with gelatin or casein. No proteolytic activity was detected either for gelatin (Figure 2B) or casein (Figure 2C). As the gel is incubated with buffer containing essential cofactors, proteolytic enzymes are able to degrade the substrate within the gel. If there is activity, proteolytic zones become visible after staining. Such zones were only visible for the positive control.

### 3.3 Assays for detection of serine- and metalloproteases

Additional highly sensitive assays were carried out to specifically detect serine proteases and metalloproteases. Samples utilized for these assays were clarified lysate and a partially purified fraction from a Q-Sepharose containing 0.2 M NaCl, where degradation was observed. For the serine protease detection, the samples were prepared to contain 5.0, 0.5, and 0.05 μg of rPspA-PdT per well. Absorbance values at 405 nm measured after incubation with L-BAPNA substrate are shown in Figure 2D. Sample values did not exceed 10% of the positive control absorbance, indicating no serine protease activity was detected by this method.

For the metalloprotease detection, the same samples and dilutions were used, corresponding to 5.0, 0.5, and 0.05 μg of rPspA-PdT per well. Figure 2E shows relative fluorescence units (RFU) measured over time (min). It is possible to observe the metalloprotease jararhagin degrading the substrate linearly over time (positive control). However, the samples maintained their RFUs between −13 and 52 units, oscillating around the noise level. Thus, no metalloproteinase activity was detected in the samples by this method.

In conclusion, no protease activity was detected by any of the four methods we carried out, leading to different strategies to investigate how and why the fusion protein was degrading.

### 3.4 Identification of cleavage site

Degraded rPspA-PdT protein had its fragments separated by IMAC-Sepharose column charged with Ni^2+^. As this hybrid protein has a His-tag at the N-terminus, the C-terminal portion resulting from degradation was collected in the flowthrough fraction of metal affinity chromatography, while the remaining protein and the N-terminal portion resulting from degradation were collected in the elution fraction with 250 mM imidazole. Flowthrough was loaded onto HPLC and the major peak fraction was collected, concentrated, and subjected to N-terminal sequencing by Edman degradation. Sequencing revealed the main cleavage site was between the two proteins rPspA and rPdT, because the N-terminal portion of the main HPLC peak was identified as the first amino acids of PdT primary sequence (LEMANKAVND, where “LE” are the amino acid residues derived from *Xho*I cloning site and “MANKAVND” are the residues coming from PdT sequence). These results led to the strategy of inserting molecular linkers between the antigens to increase stability and overcome this issue. As stability is required for licensing a new vaccine, this construct was not evaluated in further experiments.

### 3.5 Construction of genes with molecular linkers

**FIGURE 3:**
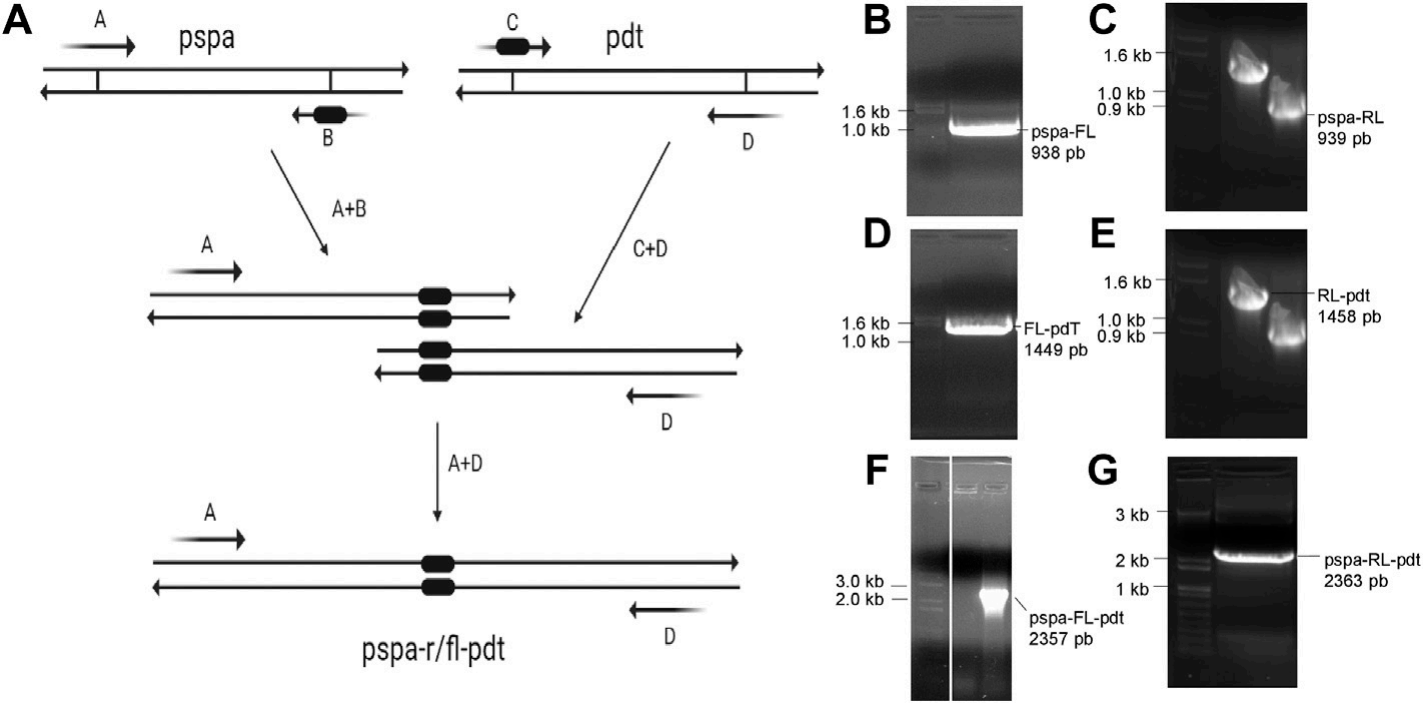
Overlap extension PCR scheme and agarose gels with PCR products. **(A)** Mechanism of molecular linker insertion by overlap extension PCR. The primers **(A–D)** used in each reaction are shown in Table 1. **(B, C)** Fragments of *pspa* amplified with a part of flexible linker (FL) and rigid linker (RL), respectively. **(D, E)** Fragments of *pdt* amplified with a part of FL and RL, respectively. **(F, G)** PCR product after amplification of complete *pspa-FL-pdt* and *pspa-RL-pdt*, respectively.

Using overlap extension PCR (Figure 3A), two new genes were constructed: *pspa-FL-pdt*, containing a flexible molecular linker between the protein genes; and *pspa-RL-pdt*, with a rigid linker separating the antigens. Initially, two PCRs were carried out in parallel to build *pspa-FL* and *FL-pdt*, both genes with a portion of the linker sequence (Figures 3B, D); then another PCR was carried out to join the previous products and obtain the full *pspa-FL-pdt gene* (Figure 3F). The same procedure was carried out for *pspa-RL-pdt* (Figures 3C, E, G). The genes were inserted in pET28a and *E. coli* strains transformed with the respective expression vectors.

### 3.6 Production and purification of new hybrid proteins with linkers

**FIGURE 4:**
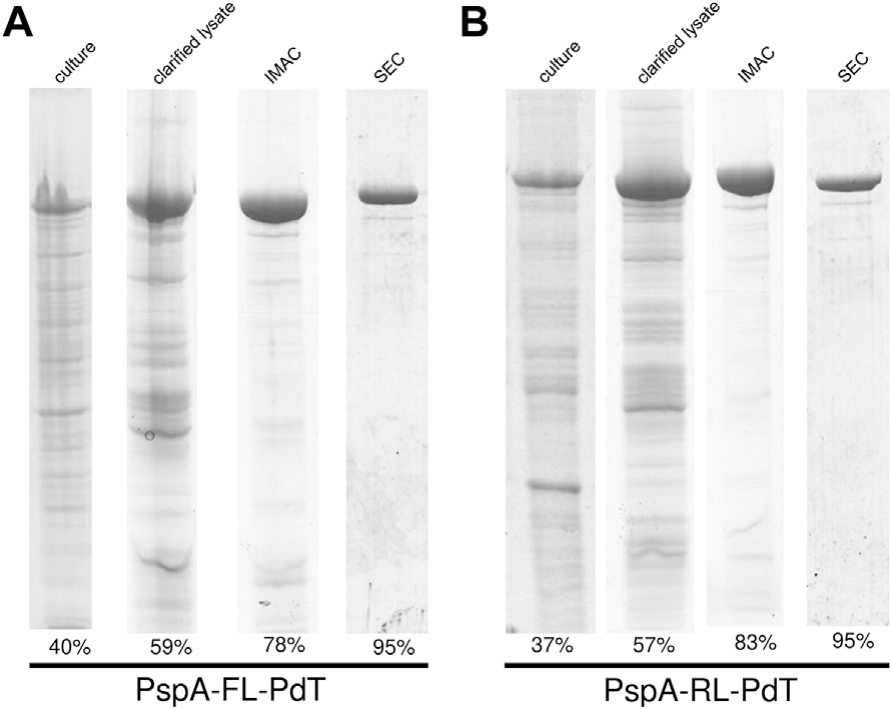
SDS-PAGE and gel densitometry for analysis of each purification step of the fusion proteins. **(A)** rPspA-FL-PdT purification steps: total protein from induced culture (40% purity), soluble fraction of clarified lysate (59%), elution of IMAC (78%) and elution of SEC (95%). **(B)** rPspA-RL-PdT purification steps: total protein from induced culture (37% purity), soluble fraction of clarified lysate (57%), elution of IMAC (83%) and elution of SEC (95%).

Hybrid proteins with linkers were produced from the transformed *E. coli* strains in chemically defined medium, pellets were lysed, incubated with 0.1% CTAB, centrifuged and proteins were purified by IMAC and size exclusion chromatography (SEC). Analysis of each step was carried out by SDS-PAGE for rPspA-FL-PdT (Figure 4A) and rPspA-RL-PdT (Figure 4B); relative quantity of fusion proteins on each fraction is described in band percentage. Elution of SEC was filtered through 0.22 µm membrane and stored at −20°C until mice immunization.

### 3.7 Stability of new hybrid proteins

**FIGURE 5:**
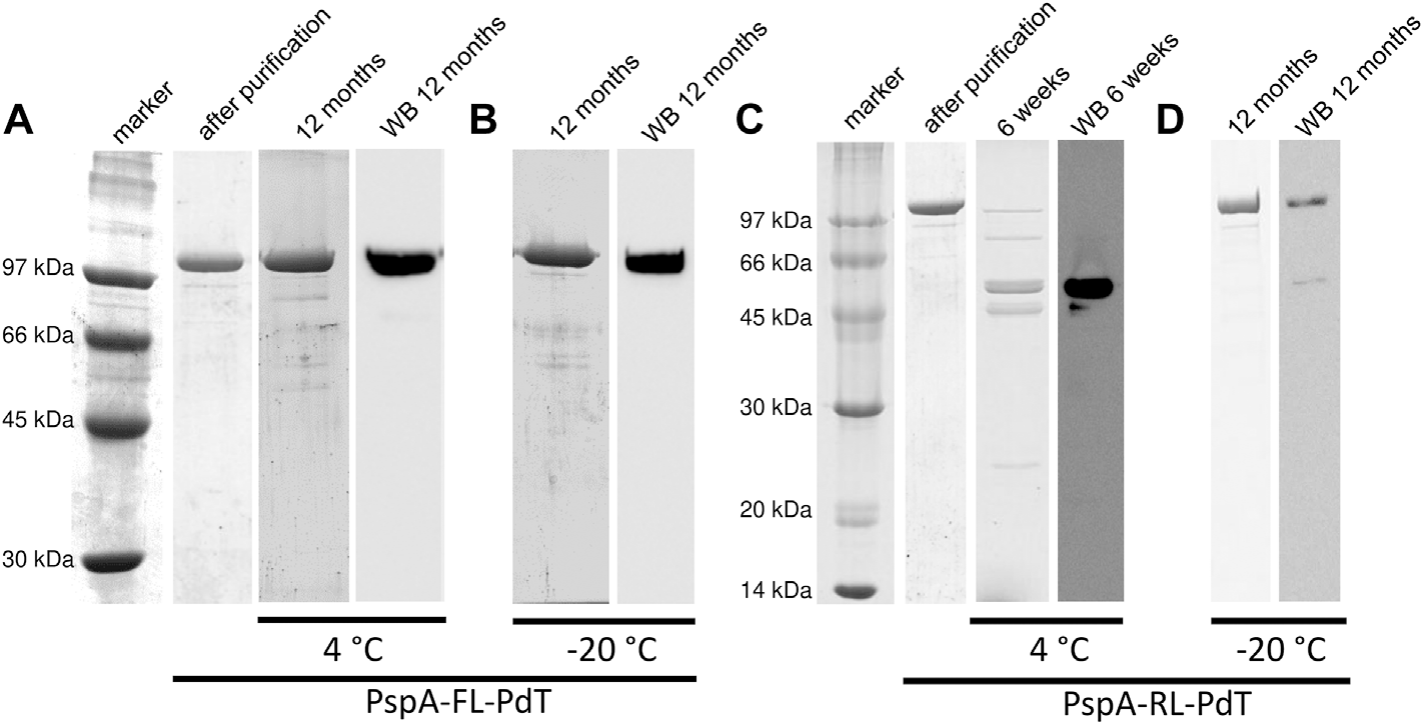
Stability periodic evaluation of the fusion proteins by SDS-PAGE and Western blot (WB). **(A, B)** rPspA-FL-PdT stored at 4°C and −20°C, respectively. rPspA-FL-PdT was evaluated just after purification (1 day) and periodically up to 12 months after purification. **(C, D)** rPspA-RL-PdT stored at 4°C and −20°C, respectively. rPspA-RL-PdT was evaluated just after purification (1 day) and periodically up to 12 months after purification; this fusion protein, however, was mostly degraded after 6 weeks stored at 4°C.

Purified proteins were sterile filtrated, maintained in Na-phosphate buffer saline (pH 7.4) at 4°C or −20°C, and evaluated periodically by SDS-PAGE and Western blot. Purified rPspA-FL-PdT was stable for at least 12 months at both 4°C (Figure 5A) and −20°C (Figure 5B). rPspA-RL-PdT degraded after 6 weeks storage at 4°C (Figure 5C). Although SDS-PAGE showed that rPspA-RL-PdT remained stable at −20°C for at least 12 months, Western blot indicated the protein underwent partial degradation (Figure 5D). High performance size exclusion chromatography also assessed the stability of the fusion proteins by comparing peak %area, using 18-month sterile filtrated purified samples stored in Na-phosphate buffer saline (pH 7.4) at 4°C or −20°C. In the case of rPspA-FL-PdT stored at −20°C, the fusion protein peak corresponded to 97.1% of total area, while another peak detected showed 2.9% (Supplementary Figure S5). When rPspA-FL-PdT was stored at 4°C, the main peak decreased to 87.6%, and few other peaks were detected, the major one representing 9.15% of total chromatogram area (Supplementary Figure S6). For rPspA-RL-PdT stored at −20°C for the same time, the main peak representing the fusion protein showed 91.6% of total chromatogram area, and other two peaks were detected, representing 4.6% and 3.85% (Supplementary Figure S7). When rPspA-RL-PdT was stored at 4°C for 18 months, the fusion protein peak corresponded to only 38.6% of total chromatogram area (Supplementary Figure S8).

### 3.8 Circular dichroism spectra and secondary structures

**FIGURE 6:**
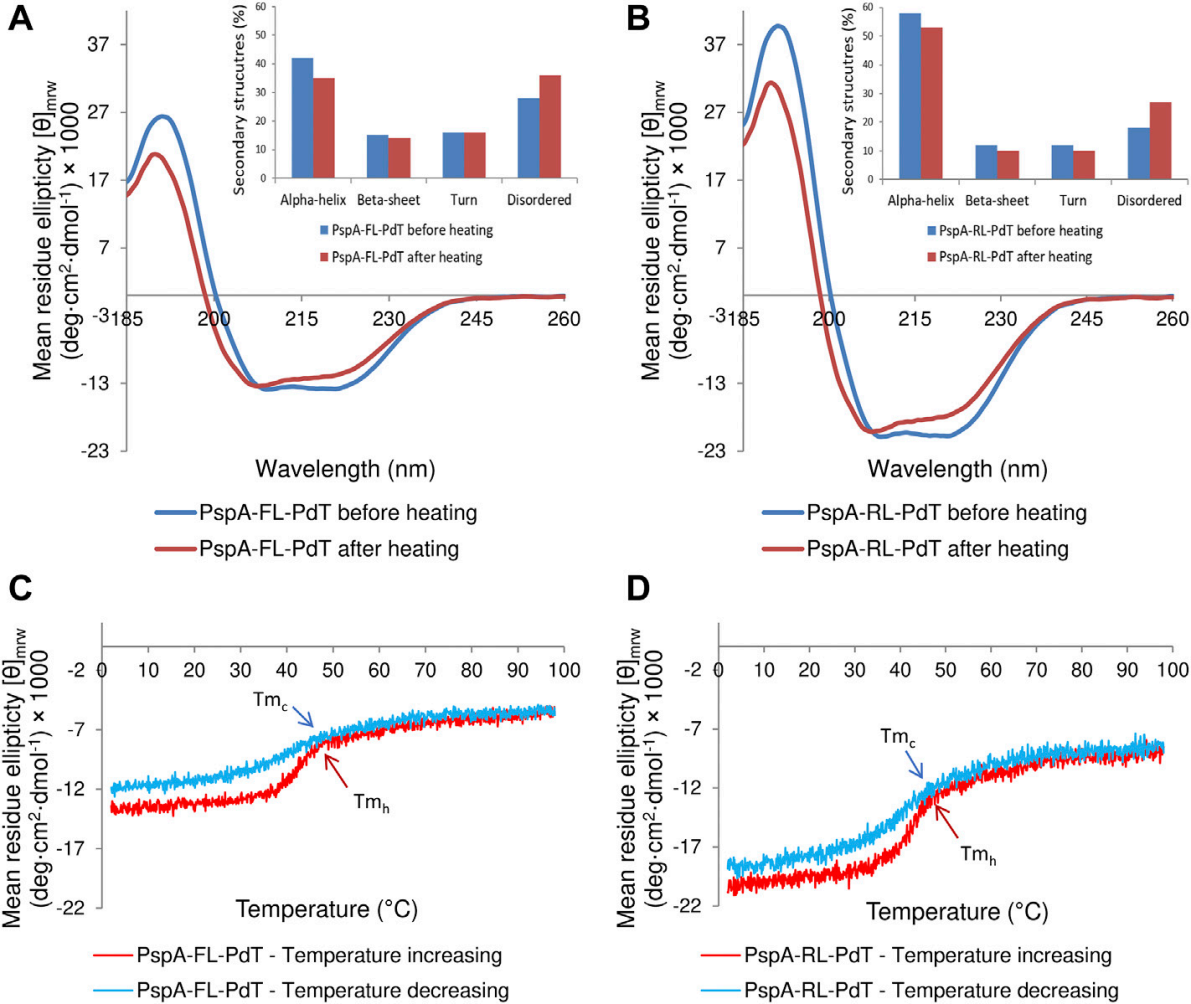
Circular dichroism spectra obtained for rPspA-FL-PdT and rPspA-RL-PdT. **(A, B)** CD spectra from 185 to 260 nm for rPspA-FL-PdT and rPspA-RL-PdT, respectively. The inset graphs show the percentage of each secondary structure before and after heating, estimated by the deconvolution of spectra using the CDSSTR algorithm. **(C, D)**. Thermal treatment of rPspA-FL-PdT and rPspA-RL-PdT, respectively, showing heating and cooling curves obtained at 222 nm from 2°C to 98°C. rPspA-FL-PdT Tm_h_ = 44.5°C and Tm_c_ = 41.81°C; rPspA-RL-PdT Tm_h_ = 44.27°C and Tm_c_ = 41.92°C.

Purified rPspA-FL-PdT and rPspA-RL-PdT were analyzed by circular dichroism spectra to determine the secondary structures before and after heating the samples (Figures 6A, B, respectively). In both spectra before heating, two valleys were observed (at approximately 208 nm and 220 nm), suggesting an alpha-helix-rich structure, which is expected for the PspA fragment in the hybrid molecule (Jedrzejas et al., 2001; Carvalho et al., 2012; Figueiredo et al., 2017). The complete spectra obtained after heating showed similar shapes but suggested a discrete loss of secondary structure. This was confirmed by deconvolution, by the percentage of secondary structures before and after heating (Figures 6A, B). β-sheets, absent in the PspA structure, were identified because they are present in the pneumolysin structure (Lawrence et al., 2015; Marshall et al., 2015). The heating and cooling curves obtained at 222 nm (Figures 6C, D), showed that the proteins lose part of their organized structures during heating, but regain part of them when cooled back. The melting temperature (Tm), at which the protein loses half of its original structure, was calculated and both hybrid molecules showed very similar values (Figures 6C, D), suggesting the difference observed regarding stability is not due to their Tm. rPspA-PdT without linker was not analyzed since the protein was unstable.

### 3.9 Tertiary structure prediction

**FIGURE 7:**
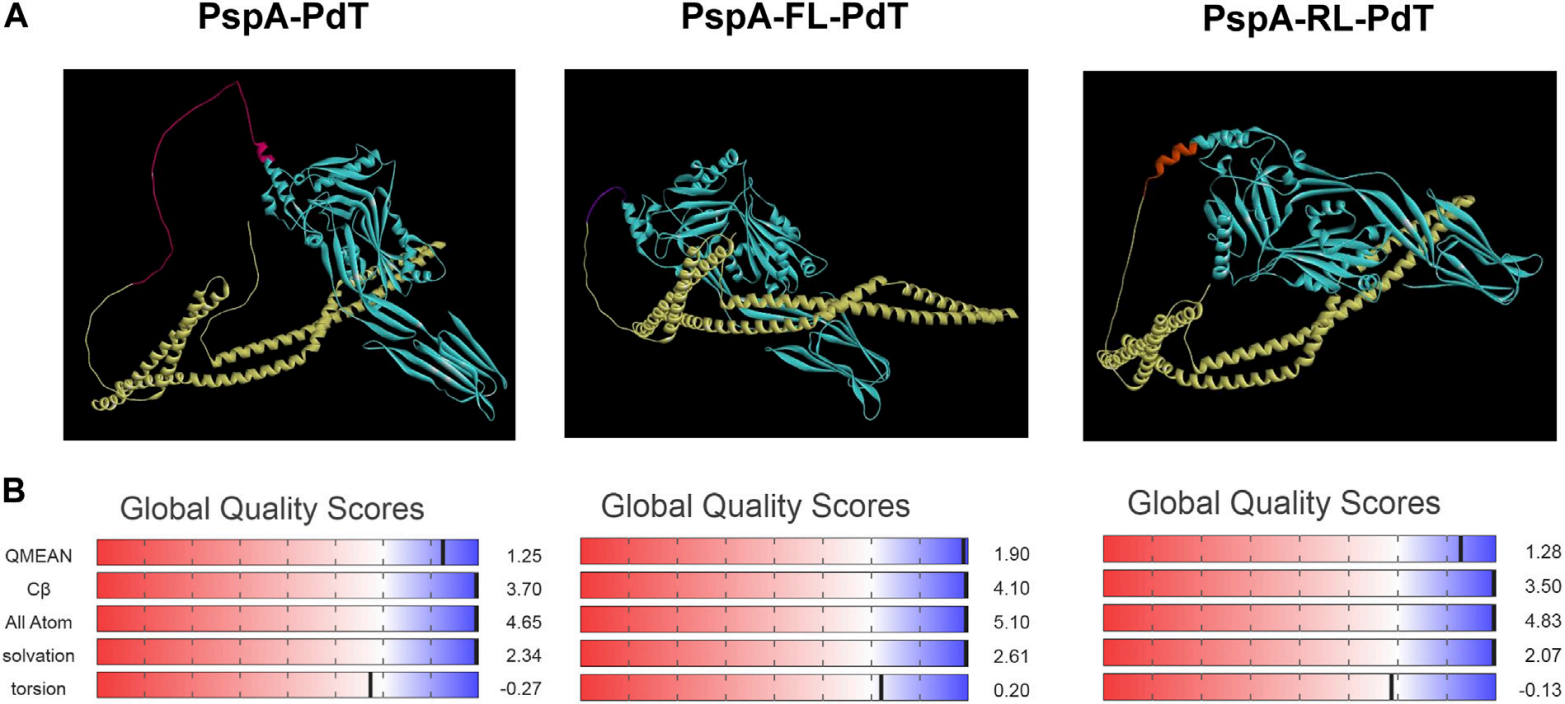
Predicted three-dimentional models of the hybrid proteins predicted by AlphaFold2 and visualized in Discovery Studio Visualizer **(A)**, and model quality estimation by QMEAN scores **(B)**. PspA is shown in yellow and PdT, in cyan **(A)**. Other colors in the models correspond to the following: for PspA-PdT, the indicated area in pink presented instability in previous works and was removed for the new constructions; for PspA-FL-PdT, flexible linker between the proteins is shown in purple; for PspA-RL-PdT, rigid linker (alpha-helix forming) between the proteins is shown in orange.

**FIGURE 8:**
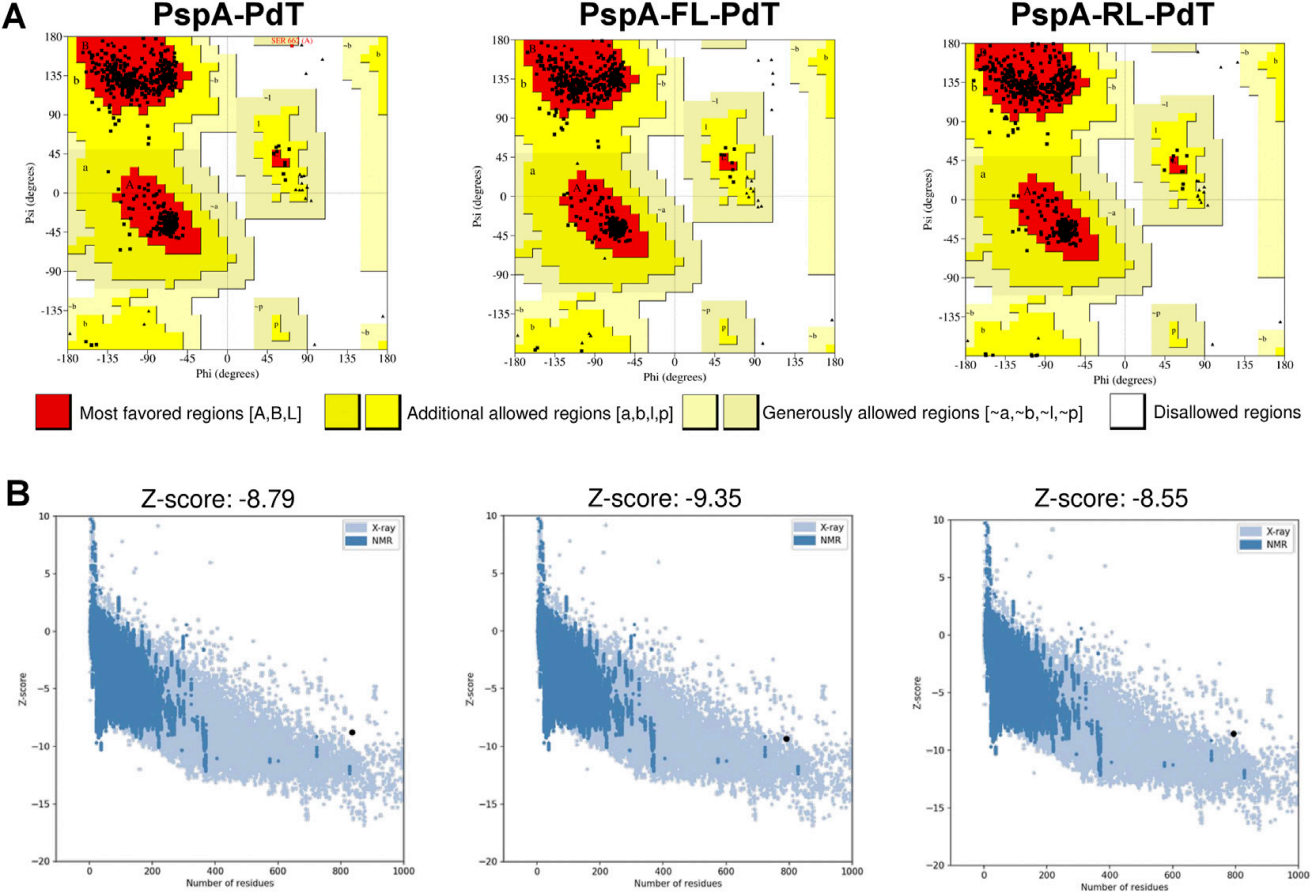
Overall model quality evaluation by PROCHECK and ProSA-web of the three hybrids. **(A)** Ramachandran plots display the percentage of residues in most favored (red), allowed (yellow) and disallowed (white) regions; respectively, for PspA-PdT: 95.7, 4.2% and 0.1%; for PspA-FL-PdT: 96.4, 3.6% and 0%; for PspA-RL-PdT: 95.5, 4.5% and 0%. Glycine residues are not counted in allowed or disallowed regions in Ramachandran plot, thus non-glycine residues are shown as black squares, and glycine residues are shown as black triangles. **(B)** Estimation of total quality by Z-score, which should not be outside the range of solved protein structures, indicating possible errors in the structures. The Z-scores of fusion proteins are shown as black circles in the graphs. It is possible to observe PspA-PdT and PspA-RL-PdT slightly outside the range, while PspA-FL-PdT falls within the area of solved structures (blue).

Since we could not find a clear difference in CD spectra that could justify the higher stability of rPspA-FL-PdT, we investigated the effects of linkers on the fusion protein stability using *in silico* modeling. The refined and validated models for each hybrid protein showed some features in the structure that could explain differences in stability (Figure 7A). In the rPspA-PdT model (without linker), the disordinate region indicated in pink represents the sequence that was removed in the new constructs. The removal of this sequence and addition of the rigid linker slightly altered the folding; meanwhile, the flexible linker insertion clearly changed the overall folding and seemed to alter the secondary structure surrounding its site. In the model quality estimation by QMEAN scores (Figure 7B), the highest score was obtained by rPspA-FL-PdT, while rPspA-PdT scored a more distant value and the scores for rPspA-RL-PdT remained almost the same for the tandem fusion protein. Analyzing the Ramachandran plots (Figure 8A), rPspA-PdT was the only model that presented residues in disallowed regions, and rPspA-FL-PdT showed more residues in the most favored regions. rPspA-PdT and rPspA-RL-PdT fell slightly off the Z-score range for solved native protein structures (Figure 8B).

### 3.10 Induction of specific antibodies by immunization

**FIGURE 9:**
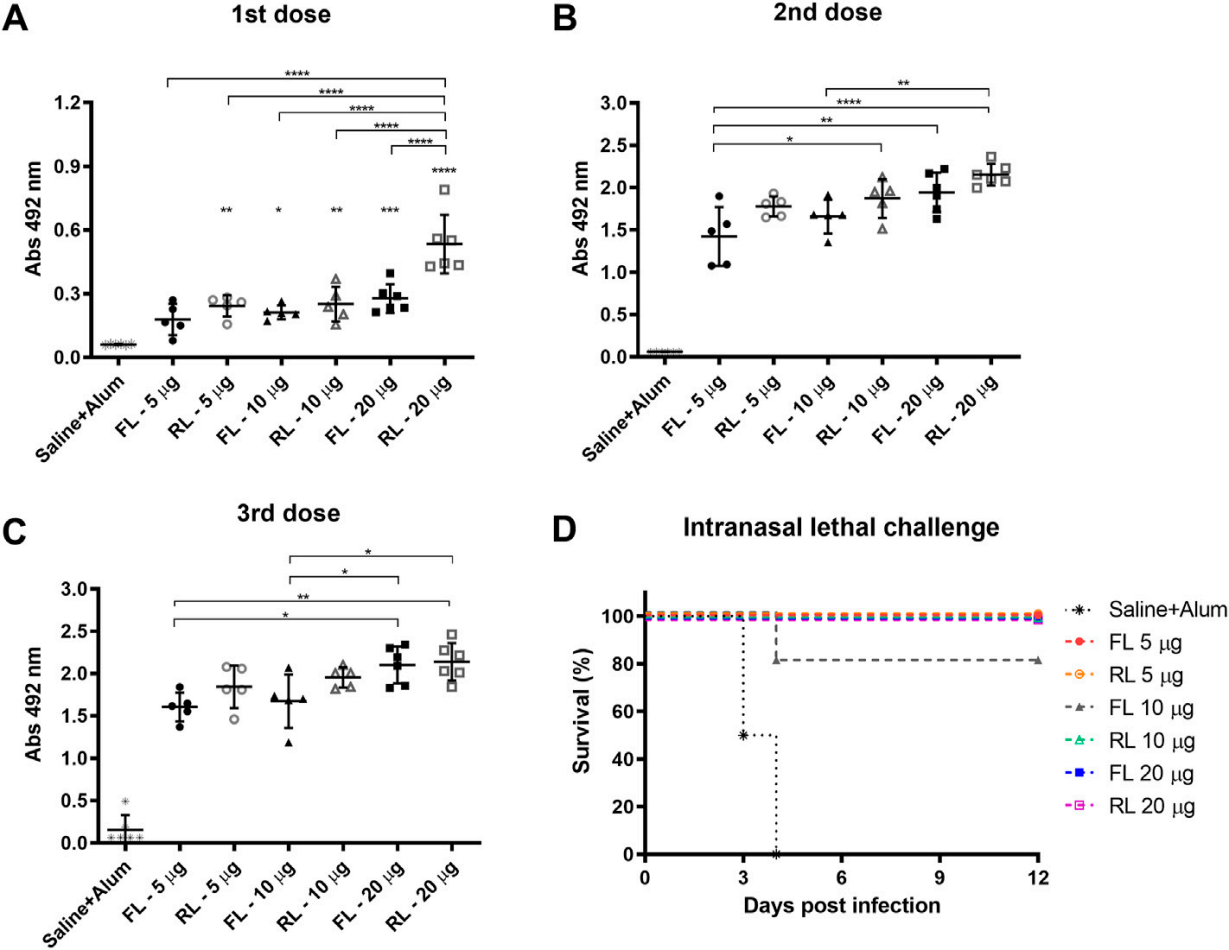
Induction of specific IgG anti-rPspA-FL-PdT and anti-rPspA-RL-PdT by subcutaneous immunization and survival curves after pneumococcal intranasal lethal challenge. Mice were immunized with three doses of 5 μg, 10 μg or 20 μg of rPspA-FL-PdT or rPspA-RL-PdT. The animals were immunized with 15-days interval with the indicated masses or control containing only the adjuvant Alum (50 μg) in saline. Blood was collected 14 days after each immunization and analyzed by ELISA. **(A–C)** Antibody levels after first, second and third immunization, respectively, are indicated by absorbance at 492 nm of 16,000-fold diluted sera. Statistically significant differences are indicated (One-way ANOVA, Tukey’s Multiple Comparison Test). *p < 0.05, **p < 0.01, ***p < 0.001, ****p < 0.0001. For B and C, the difference in relation to the control group is not indicated, as it presented *p* < 0.0001 compared to all other groups. **(D)** Survival curves of mice after intranasal lethal challenge with *S. pneumoniae* strain A66.1. Five immunized groups showed 100% of protection, and one immunized group showed 80%. Animals were challenged with 1 × 10^5^ CFU intranasally 21 days after the third dose. Curves were analyzed by the Log-rank test (Mantel-Cox).

**FIGURE 10:**
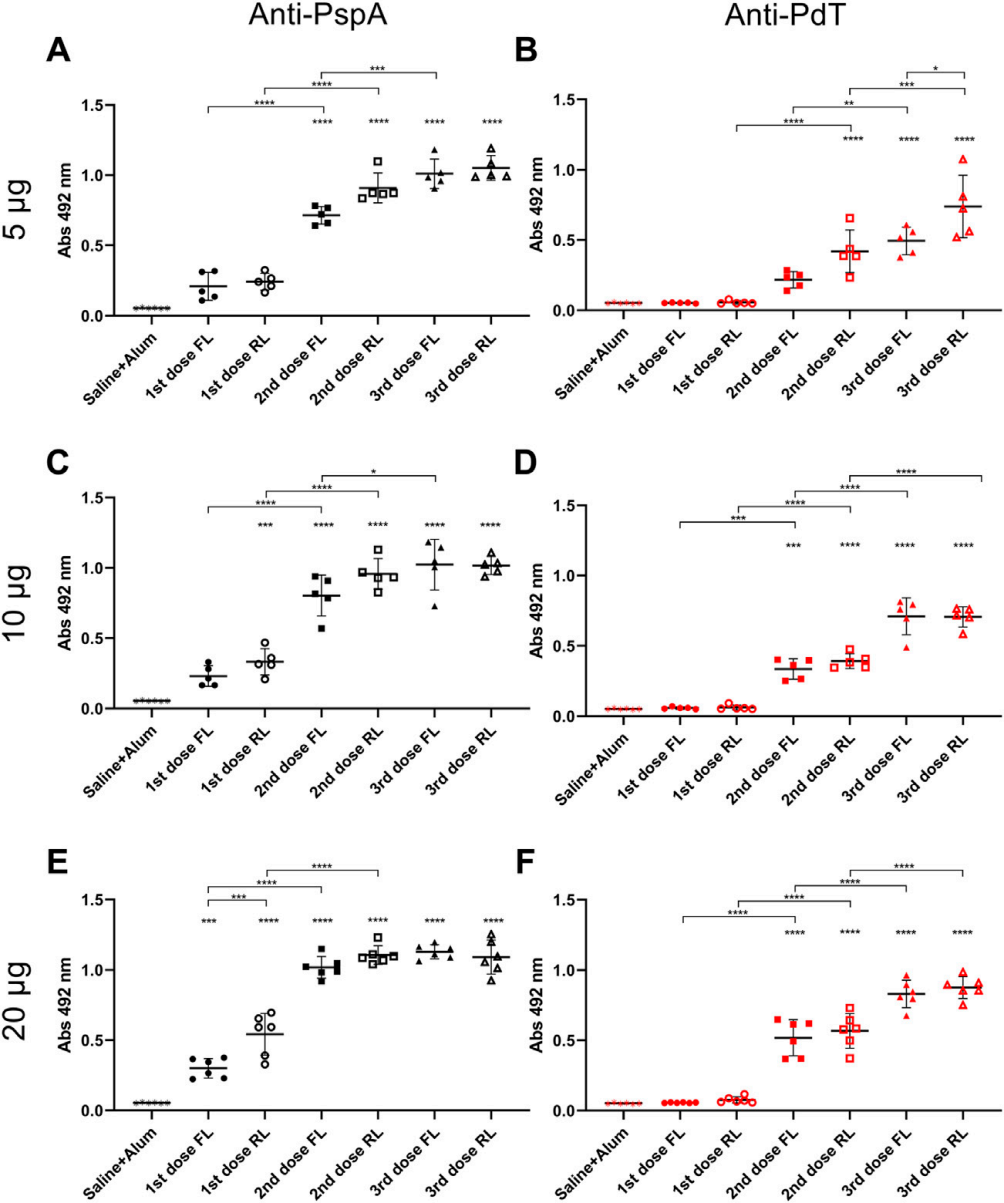
Induction of specific IgG anti-rPspA and anti-rPdT by subcutaneous immunization. Mice were immunized with three doses of 5 μg **(A, B)**, 10 μg **(C, D)** or 20 μg **(E, F)** of rPspA-FL-PdT or rPspA-RL-PdT. The animals were immunized within 15-day interval with the indicated masses or control containing only the adjuvant Alum (50 μg) in saline. Blood was collected 14 days after each immunization and analyzed by ELISA. Antibody levels are indicated by absorbance at 492 nm of 4,000-fold diluted sera. Statistically significant differences are indicated (One-way ANOVA, Tukey’s Multiple Comparison Test). *p < 0.05, **p < 0.01, ***p < 0.001, ****p < 0.0001. Differences in relation to the control group is indicated on top of the groups and brackets above groups indicate difference between groups.

BALB/c mice received three subcutaneous immunizations with 15-day interval containing different doses of rPspA-FL-PdT or rPspA-RL-PdT in sterile PBS with 50 µg Alum; control groups received only Alum with 0.9% sterile saline. Specific IgG antibody levels were measured 15 days after each immunization. Statistical differences between the two different fusion proteins (rPspA-FL-PdT and rPspA-RL-PdT) and the different doses (5 μg, 10 μg and 20 μg) were compared (Figures 9A–C). Initially, the groups were compared only within each immunization. Then, the first, second and third immunizations within each of the groups were also compared. While the difference between the first and second immunization was notable (*p* < 0.0001) for all groups, there was no statistically significant difference between the second and third doses. Although vaccination with rPspA-RL-PdT led to higher antibody production when compared with rPspA-FL-PdT groups of the same doses, the differences were not considered statistically significant (except for FL20 versus RL20 after the first immunization, *p* < 0.0001). While there was no statistical differences between the second and third doses regarding the antibodies against the entire fusion proteins, we could observe interesting differences for the antigens separately (Figure 10). In general, the results regarding IgG anti-PspA are similar to the antibody levels against the entire fusion proteins, i.e., differences are much more evident comparing the first and second doses, and a plateau is reached after the second dose (Figures 10A, C, E). The third dose elicited significantly higher antibodies anti-PspA only for groups immunized with 5 μg and 10 µg of rPspA-FL-PdT (Figures 10A, C), while for 20 µg or for rPspA-RL-PdT in any amount, the increase of antibody levels was not significant (Figure 10E). However, we could observe remarkable differences when comparing IgG levels anti-PdT (Figures 10B, D, F). The only difference that was not statistically significant was observed in the group immunized with 5 µg of rPspA-FL-PdT when comparing the first and second doses (Figure 10B).

### 3.11 Protection against intranasal lethal challenge

Subcutaneous immunization with rPspA-FL-PdT or rPspA-RL-PdT was also evaluated regarding its ability to protect against intranasal lethal challenge with pneumococcal strain A66.1 delivered to the lungs 21 days after the third dose. All immunized mice, except one in the group that received 10 μg of rPspA-FL-PdT, survived the challenge. Comparing the survival curve (Figure 9D) of the control group to all other groups (except FL10), the difference was significant (*p* = 0.002); comparison between control and FL10 was also significant (*p* = 0.0071); and comparison between FL10 and the other groups was not considered statistically different (*p* = 0.3173). The only immunized animal that did not survive showed marked piloerection and reduced mobility, being euthanized on the fourth day. All other immunized animals showed no apparent clinical signs of infection.

## 4 Discussion

Recombinant fusion proteins have been used as a versatile tool for a wide range of applications, providing a useful strategy not only for increasing stability, expression or purification yield, but also for enabling the production of multiple functional and synergistic protein domains in a single process (Chen et al., 2013; Yu et al., 2015). Some fusion biopharmaceutical proteins are available in the market and represent a substantial part of the drug market, such as Ontak^®^(IL-2 and denileukin diftitox for cutaneous T-cell lymphoma) and Idelvion^®^ (Factor IX and albumin for hemophilia B). Even COVID-19 vaccines are being developed using fusion proteins, such as NVX-CoV2373 (Novavax) (Dunkle et al., 2022), which uses recombinant spike protein trimmers; and S-Trimer (Clover Biopharmaceuticals), genetical fusion of SARS-CoV-2 S-protein to human C-propeptide of alpha1(I) collagen (Liang et al., 2020), promoting a self-trimerization and enabling purification *via* affinity chromatography. Pollet et al. (2021) highlight the stability of the vaccine at refrigerated temperatures (2°C–8°C), an important point to address ongoing epidemics, especially for middle and low-income countries (Crommelin et al., 2021), places where the pneumococcal burden is the highest (Wahl et al., 2018).

Ideally, the fusion of proteins should include physico-chemically compatible molecules and their position inside the hybrid molecule should be carefully chosen. In general, the straightforward method would be tandem fusion, where the genes are combined end-to-end and the result is a single polypeptide chain (Yu et al., 2015). In the case of two proteins, both options of order should be tested unless there is a hypothesis for a specific order. In our case, this specific order was chosen because PspA is a well-known soluble protein (Figueiredo et al., 2017; Cardoso et al., 2022), which may act as a solubility enhancer for PdT, a protein that frequently presents solubility issues in our lab (data not shown). Moreover, the PspA sequence that we selected presents a part of its proline-rich region in C-terminal, consisting of several repeated residues of proline and alanine that could act as a “bridge” between protein domains, since some described rigid linkers are composed of these residues (Zhao et al., 2008).

The end-to-end strategy did not provide suitable stability to the hybrid molecule. Within the proline-rich region, the PspA sequence also presents a non-proline sequence block (NonPro), which decreased stability and caused the sequence breaking down mostly between the molecules, as identified by N-terminal sequencing by Edman degradation method. Removal of the NonPro region not only improves stability, but also could expand immunogenicity: anti-rPspA antibodies induced by rPspAs lacking the NonPro region showed enhanced ability to bind to pneumococcal strains with PspAs that do not contain NonPro (Moreno et al., 2010). Since not all pneumococcal strains contain NonPro (Hollingshead et al., 2000), a vaccine that does not comprise NonPro PspA could present higher coverage.

The proteins joined in tandem, thus, resulted in unfavored folding and scored low in both stereochemistry (residues in disallowed regions according to Ramachandran plot) and Z-score (model outside the range of solved native protein structures), which may indicate not only problems with purification but also production and bioactivity. Although the immunization with rPspA-PdT without linker showed the best results in comparison to PspA and PdT, alone or co-administrated (Goulart et al., 2013), the stability issues impaired a successful scale up of the production. As stability is crucial for bioprocess development of biopharmaceutical products, we decided not to investigate this fusion protein further.

Several investigations achieved enhanced stability, expression and activity by inserting flexible or rigid linkers between protein domains. Lu and Feng (2008) constructed bifunctional fusions of β-glucanase and xylanase with an expressive increase in activity when using (GGGGGS)_3_ and (EAAAK)_3_ linkers between the moieties, especially regarding the flexible spacer. In general, rigid or helical linkers are expected to effectively separate fusion proteins domains (Arai et al., 2001; Bai and Shen, 2006), and thus preserving their independent functions. However, for some proteins, the flexibility given by glycine and serine linkers is desirable, such as the “fold-back” conformation that the single-chain variable fragment (scFv) antibody acquired when a long flexible linker (GGGGS)_3_ allowed interactions between domains and increased binding (Kim et al., 2007). Also, Hu et al. (2004) introduced (GGGGS)_3_ to link two copies of HBsAg preS1 fused to glutathione S-transferase and much stronger immunoreactivity was achieved than the same molecules without linkers. Flexible linkers can also be used to link two monomers in order to produce a stable dimer (Minshawi et al., 2020).

In this work, experimental data had shown differences in the stability of fusion proteins when using flexible or rigid linker between PspA and PdT; however, these data, together with circular dichroism spectra, had not elucidated how the linkers were affecting stability. Therefore, we used *in silico* analyses to search for possible mechanisms. Experimental data showed increased stability provided by the flexible linker, and *in silico* analyses showed better Z-scores for rPspA-FL-PdT, suggesting that in this case the hybrid molecule overall stability is favored when the domains were separated by this linker. The linker effects vary from protein to protein and can be analyzed depending on the desired response. Zhao et al. (2008) demonstrated that all linkers tested (flexible, helical and rigid, composed of prolines and alanines) were effective in separating human serum albumin and interferon-α2b; however, each one was better in a specific response: while helical linker increased anti-viral activity the most, it was more susceptible to hydrolysis during stability tests, thus making it a hard decision when choosing the best spacer. Stability must play a major role in a vaccine formulation, especially against pneumococcus and other diseases that affect mainly developing countries. Lowering production costs is important, but also it must be taken in consideration the wide-reaching distribution network; for example, formulations that require storage at ultra-cold freezers will most likely reduce the access for emerging economies and the poorest countries (Frati et al., 2022).

The ability of the hybrid proteins to protect mice against pneumococcal lethal challenge was evaluated by intranasal challenge 21 days after the third immunization. The amount of protein used for the immunization presented some significant differences among groups in antibody levels against the entire fusion proteins, however, there was no significant difference regarding the linker used or between second and third doses. On the other hand, the IgG levels anti-PdT after the third dose were significantly higher than after the second dose in all groups. This is especially important since pneumolysin is a more conserved protein than PspA among *S. pneumoniae* strains, thus the fusion protein could potentially offer broader protection against different strains after three doses.

In addition, no significant differences were observed in the protection achieved using both fusion proteins and all groups were protected against pneumococcal lethal challenge. Park et al. (2017) challenged BALB/c mice with 1 × 10^5^ CFU of *S. pneumoniae* A66.1, the same strain used in this work, and mentioned that, to assess protective immunity, the lethal dose used was 100 times the LD_50_; the dose used in the present study was, therefore, adequate to assess protective immunity. In some preclinical studies using pneumococcal proteins and adjuvants, it was possible to achieve 100% protection, although rare. Survival after immunization with rPspA ranged from 25% to 60% and with PspC, 0%–20% (Ferreira et al., 2009). Ogunniyi et al. (2007) tested several proteins and their combinations and challenged with different pneumococcal and mice strains; they highlighted that relative protection obtained by single proteins is strain dependent and demonstrated that nearly all combinations of antigens provided higher protection than the proteins alone, which was also observed previously (Ogunniyi et al., 2000; Briles et al., 2003). This is also the case for our paper that originated the present work (Goulart et al., 2013), which showed higher protection with rPspA and rPdT than with each protein alone. Oliveira et al. (2010) showed protection of 40%–50% from rPspA alone, increasing to 90%–100% when administered with whole-cell pertussis vaccine as adjuvant. The Alum adjuvant, used here, helps to maintain higher circulating levels of antibodies for a longer time in relation to an antigen inoculated only in aqueous solution (Jafari et al., 2017). Good results obtained with the antigens alone may be an indication that they will be even better when formulated with novel delivery technologies such as nanoparticles, nanogels and bacterial or viral-like particles (BLPs and VLPs, respectively), generating robust humoral and cellular responses (Gonçalves et al., 2019).

Some protein vaccines developed against pneumococcus have successfully undergone phase 1 and 2 clinical trials, however, no vaccine other than capsular polysaccharide-based, whether conjugated (PCV) or not, has been in a phase 3 and licensed (Oliveira et al., 2021). PCVs have a proven track record of success in reducing pneumococcal burden (Wahl et al., 2018) despite their limitations, and are already under constant improvement by big pharmaceutical companies; the development plan is known and established. Alternative vaccines not based in capsular polysaccharides have no immunological correlates of protection defined to compare clinical relevance of the trials (Prymula et al., 2014). Protein-based and other technologies may not correlate with opsonophagocytic assay, the gold-standard for PCVs, but to other assays yet to be defined (Pichichero, 2017). Establishing these correlates is a very important step to include protein vaccines in the radar for the big companies and to raise funds for expensive clinical trials such as phase 3. PCVs valency keeps increasing (Huang et al., 2022; Platt et al., 2022) to address serotype replacement and coverage limitations, but also does the complexity involved in the production process, which decreases the access for developing countries. Despite many challenges, the most effective alternative against pneumococcal burden worldwide remains establishing serotype-independent vaccines based on protein antigens (Converso et al., 2020). rPspA-PdT fusion protein joined by flexible linker, rPspA-FL-PdT, showed remarkable stability and protection against lethal challenge in mice, making it promising serotype-independent candidate for future vaccine formulations. In addition, rPspA-FL-PdT production enables to obtain two antigens in a single process, which is advantageous from bioprocessing and economic points of view. Therefore, further studies should be done to enrich the comprehension of the immune response and to improve the production process.

## Data Availability

The datasets presented in this study can be found in online repositories. The names of the repository/repositories and accession number(s) can be found in the article/Supplementary Material.

## References

[B1] Arai R. (2021). Design of helical linkers for fusion proteins and protein-based nanostructures. Methods Enzymol. 647, 209–230. 10.1016/BS.MIE.2020.10.003 33482989

[B2] Arai R. Ueda H. Kitayama A. Kamiya N. Nagamune T. (2001). Design of the linkers which effectively separate domains of a bifunctional fusion protein. Protein Eng. Des. Sel. 14, 529–532. 10.1093/protein/14.8.529 11579220

[B3] Bai Y. Shen W. C. (2006). Improving the oral efficacy of recombinant granulocyte colony-stimulating factor and transferrin fusion protein by spacer optimization. Pharm. Res. 23, 2116–2121. 10.1007/s11095-006-9059-5 16952003

[B4] Barazzone G. C. Carvalho R. Kraschowetz S. Horta A. L. Sargo C. R. Silva A. J. (2011). Production and purification of recombinant fragment of pneumococcal surface protein A (PspA) in *Escherichia coli* . Procedia Vaccinol. 4, 27–35. 10.1016/J.PROVAC.2011.07.005

[B5] Benkert P. Künzli M. Schwede T. (2009). QMEAN server for protein model quality estimation. Nucleic Acids Res. 37, W510–W514. 10.1093/NAR/GKP322 19429685 PMC2703985

[B6] Berry A. M. Alexander J. E. Mitchell T. J. Andrew P. W. Hansman D. Paton J. C. (1995). Effect of defined point mutations in the pneumolysin gene on the virulence of *Streptococcus pneumoniae* . Infect. Immun. 63, 1969–1974. 10.1128/iai.63.5.1969-1974.1995 7729909 PMC173251

[B7] Braun J. S. Sublett J. E. Freyer D. Mitchell T. J. Cleveland J. L. Tuomanen E. I. (2002). Pneumococcal pneumolysin and H(2)O(2) mediate brain cell apoptosis during meningitis. J. Clin. Invest. 109, 19–27. 10.1172/JCI12035 11781347 PMC150815

[B8] Briles D. E. Hollingshead S. Brooks-Walter A. Nabors G. S. Ferguson L. Schilling M. (2000a). The potential to use PspA and other pneumococcal proteins to elicit protection against pneumococcal infection. Vaccine 18, 1707–1711. 10.1016/S0264-410X(99)00511-3 10689153

[B9] Briles D. E. Hollingshead S. K. King J. Swift A. Braun P. A. Park M. K. (2000b). Immunization of humans with recombinant pneumococcal surface protein A (rPspA) elicits antibodies that passively protect mice from fatal infection with *Streptococcus pneumoniae* bearing heterologous PspA. J. Infect. Dis. 182, 1694–1701. 10.1086/317602 11069242

[B10] Briles D. E. Hollingshead S. K. Paton J. C. Ades E. W. Novak L. Van Ginkel F. W. (2003). Immunizations with pneumococcal surface protein A and pneumolysin are protective against pneumonia in a murine model of pulmonary infection with *Streptococcus pneumoniae* . J. Infect. Dis. 188, 339–348. 10.1086/376571 12870114

[B11] Bryksin A. V. Matsumura I. (2010). Overlap extension PCR cloning: A simple and reliable way to create recombinant plasmids. Biotechniques 48, 463–465. 10.2144/000113418 20569222 PMC3121328

[B12] Cardoso V. M. Paredes S. A. H. Campani G. Gonçalves V. M. Zangirolami T. C. (2022). ClearColi as a platform for untagged pneumococcal surface protein A production: Cultivation strategy, bioreactor culture, and purification. Appl. Microbiol. Biotechnol. 106, 1011–1029. 10.1007/s00253-022-11758-9 35024919 PMC8755982

[B13] Carvalho R. J. Cabrera-Crespo J. Tanizaki M. M. Gonçalves V. M. (2012). Development of production and purification processes of recombinant fragment of pneumococcal surface protein A in *Escherichia coli* using different carbon sources and chromatography sequences. Appl. Microbiol. Biotechnol. 94, 683–694. 10.1007/s00253-011-3649-9 22075630

[B14] Ceballos-Alcantarilla E. Merkx M. (2021). Understanding and applications of Ser/Gly linkers in protein engineering. Methods Enzymol. 647, 1–22. 10.1016/BS.MIE.2020.12.001 33482985

[B15] Chen X. Zaro J. L. Shen W. C. (2013). Fusion protein linkers: Property, design and functionality. Adv. Drug Deliv. Rev. 65, 1357–1369. 10.1016/J.ADDR.2012.09.039 23026637 PMC3726540

[B16] Clutterbuck E. A. Lazarus R. Yu L.-M. Bowman J. Bateman E. A. L. Diggle L. (2012). Pneumococcal conjugate and plain polysaccharide vaccines have divergent effects on antigen-specific B cells. J. Infect. Dis. 205, 1408–1416. 10.1093/infdis/jis212 22457293 PMC3324398

[B17] Converso T. R. Assoni L. André G. O. Darrieux M. Leite L. C. C. (2020). The long search for a serotype independent pneumococcal vaccine. Expert Rev. Vaccines 19, 57–70. 10.1080/14760584.2020.1711055 31903805

[B18] Corcoran M. Vickers I. Mereckiene J. Murchan S. Cotter S. Fitzgerald M. (2017). The epidemiology of invasive pneumococcal disease in older adults in the post-PCV era. Has there been a herd effect? Epidemiol. Infect. 145, 2390–2399. 10.1017/S0950268817001194 28712384 PMC9148822

[B19] Crommelin D. J. A. Anchordoquy T. J. Volkin D. B. Jiskoot W. Mastrobattista E. (2021). Addressing the cold reality of mRNA vaccine stability. J. Pharm. Sci. 110, 997–1001. 10.1016/J.XPHS.2020.12.006 33321139 PMC7834447

[B20] Darrieux M. Goulart C. Briles D. Leite L. C. de C. (2015). Current status and perspectives on protein-based pneumococcal vaccines. Crit. Rev. Microbiol. 41, 190–200. 10.3109/1040841X.2013.813902 23895377

[B21] Dunkle L. M. Kotloff K. L. Gay C. L. Áñez G. Adelglass J. M. Barrat Hernández A. Q. (2022). Efficacy and safety of NVX-CoV2373 in adults in the United States and Mexico. N. Engl. J. Med. 386, 531–543. 10.1056/NEJMoa2116185 34910859 PMC8693692

[B22] Edman P. Högfeldt E. Sillén L. G. Kinell P.-O. (1950). Method for determination of the amino acid sequence in peptides. Acta Chem. Scand. 4, 283–293. 10.3891/ACTA.CHEM.SCAND.04-0283

[B23] Entwisle C. Hill S. Pang Y. Joachim M. McIlgorm A. Colaco C. (2017). Safety and immunogenicity of a novel multiple antigen pneumococcal vaccine in adults: A phase 1 randomised clinical trial. Vaccine 35, 7181–7186. 10.1016/J.VACCINE.2017.10.076 29132988

[B24] Fernández-Resa P. Mira E. Quesada A. R. (1995). Enhanced detection of casein zymography of matrix metalloproteinases. Anal. Biochem. 224, 434–435. 10.1006/ABIO.1995.1063 7710105

[B25] Ferreira D. M. Darrieux M. Silva D. A. Leite L. C. C. Ferreira J. M. C. Ho P. L. (2009). Characterization of protective mucosal and systemic immune responses elicited by pneumococcal surface protein PspA and PspC nasal vaccines against a respiratory pneumococcal challenge in mice. Clin. Vaccine Immunol. 16, 636–645. 10.1128/CVI.00395-08 19279169 PMC2681601

[B26] Figueiredo D. B. Carvalho E. Santos M. P. Kraschowetz S. Zanardo R. T. Campani G. (2017). Production and purification of an untagged recombinant pneumococcal surface protein A (PspA4Pro) with high-purity and low endotoxin content. Appl. Microbiol. Biotechnol. 101, 2305–2317. 10.1007/s00253-016-7983-9 27889801

[B27] Frati P. Torre G. L. Daems R. Maes E. (2022). The race for COVID-19 vaccines: Accelerating innovation, fair allocation and distribution. Vaccines (Basel). 10, 1450. *Vaccines 2022Page 1450* 10. 10.3390/VACCINES10091450 36146528 PMC9500728

[B28] Freitas-de-sousa L. A. Colombini M. Lopes-Ferreira M. Serrano S. M. T. Moura-da-silva A. M. (2017). Insights into the mechanisms involved in strong hemorrhage and dermonecrosis induced by atroxlysin-ia, a PI-class snake venom metalloproteinase. Toxins (Basel) 9, 239. 10.3390/TOXINS9080239 28767072 PMC5577573

[B29] Ganaie F. Maruhn K. Li C. Porambo R. J. Elverdal P. L. Abeygunwardana C. (2021). Structural, genetic, and serological elucidation of *Streptococcus pneumoniae* serogroup 24 serotypes: Discovery of a new serotype, 24C, with a variable capsule structure. J. Clin. Microbiol. 59, e0054021. 10.1128/JCM.00540-21 33883183 PMC8218768

[B30] Geno K. A. Gilbert G. L. Song J. Y. Skovsted I. C. Klugman K. P. Jones C. (2015). Pneumococcal capsules and their types: Past, present, and future. Clin. Microbiol. Rev. 28, 871–899. 10.1128/CMR.00024-15 26085553 PMC4475641

[B31] Gonçalves V. M. Dias W. O. Campos I. B. Liberman C. Sbrogio-Almeida M. E. Silva E. P. (2014). Development of a whole cell pneumococcal vaccine: BPL inactivation, cGMP production, and stability. Vaccine 32, 1113–1120. 10.1016/J.VACCINE.2013.10.091 24342254

[B32] Gonçalves V. M. Kaneko K. Solórzano C. MacLoughlin R. Saleem I. Miyaji E. N. (2019). Progress in mucosal immunization for protection against pneumococcal pneumonia. Expert Rev. Vaccines 18, 781–792. 10.1080/14760584.2019.1643719 31305196

[B33] Gonçalves V. M. Takagi M. Lima R. B. Massaldi H. Giordano R. C. Tanizaki M. M. (2003). Purification of capsular polysaccharide from *Streptococcus pneumoniae* serotype 23F by a procedure suitable for scale-up. Biotechnol. Appl. Biochem. 37, 283–287. 10.1042/BA20020075 12515577

[B34] Goulart C. da Silva T. R. Rodriguez D. Politano W. R. Leite L. C. C. Darrieux M. (2013). Characterization of protective immune responses induced by pneumococcal surface protein A in fusion with pneumolysin derivatives. PLoS One 8, e59605. 10.1371/journal.pone.0059605 23533636 PMC3606166

[B35] Goulart C. Darrieux M. Rodriguez D. Pimenta F. C. Brandileone M. C. C. de Andrade A. L. S. S. (2011). Selection of family 1 PspA molecules capable of inducing broad-ranging cross-reactivity by complement deposition and opsonophagocytosis by murine peritoneal cells. Vaccine 29, 1634–1642. 10.1016/J.VACCINE.2010.12.074 21211592

[B36] Han C. Zhang M. (2019). Genetic diversity and antigenicity analysis of *Streptococcus pneumoniae* pneumolysin isolated from children with pneumococcal infection. Int. J. Infect. Dis. 86, 57–64. 10.1016/j.ijid.2019.06.025 31255709

[B37] Hargreaves J. R. Greenwood B. Clift C. Goel A. Roemer-Mahler A. Smith R. (2011). Making new vaccines affordable: A comparison of financing processes used to develop and deploy new meningococcal and pneumococcal conjugate vaccines. Lancet (London, Engl. 378, 1885–1893. 10.1016/S0140-6736(11)60687-9 21664678

[B38] Hausdorff W. P. Bryant J. Paradiso P. R. Siber G. R. (2000). Which pneumococcal serogroups cause the most invasive disease: Implications for conjugate vaccine formulation and use, part I. Clin. Infect. Dis. 30, 100–121. 10.1086/313608 10619740

[B39] Henrichsen J. (1995). Six newly recognized types of *Streptococcus pneumoniae* . J. Clin. Microbiol. 33, 2759–2762. 10.1128/jcm.33.10.2759-2762.1995 8567920 PMC228570

[B40] Henriques-Normark B. Tuomanen E. I. (2013). The pneumococcus: Epidemiology, microbiology, and pathogenesis. Cold Spring Harb. Perspect. Med. 1, 3a010215. 10.1101/cshperspect.a010215 PMC3685878 23818515

[B41] Heo L. Park H. Seok C. (2013). GalaxyRefine: Protein structure refinement driven by side-chain repacking. Nucleic Acids Res. 41, W384–W388. 10.1093/NAR/GKT458 23737448 PMC3692086

[B42] Hieng B. Ugrinović K. Šuštar-Vozlič J. Kidrič M. (2004). Different classes of proteases are involved in the response to drought of Phaseolus vulgaris L. cultivars differing in sensitivity. J. Plant Physiol. 161, 519–530. 10.1078/0176-1617-00956 15202708

[B43] Ho S. N. Hunt H. D. Horton R. M. Pullen J. K. Pease L. R. (1989). Site-directed mutagenesis by overlap extension using the polymerase chain reaction. Gene 77, 51–59. 10.1016/0378-1119(89)90358-2 2744487

[B44] Hollingshead S. K. Becker R. Briles D. E. (2000). Diversity of PspA: Mosaic genes and evidence for past recombination in *Streptococcus pneumoniae* . Infect. Immun. 68, 5889–5900. 10.1128/IAI.68.10.5889-5900.2000 10992499 PMC101551

[B45] Hu W. Li F. Yang X. Li Z. Xia H. Li G. (2004). A flexible peptide linker enhances the immunoreactivity of two copies HBsAg preS1 (21–47) fusion protein. J. Biotechnol. 107, 83–90. 10.1016/J.JBIOTEC.2003.09.009 14687974

[B46] Huang L. Wasserman M. Grant L. Farkouh R. Snow V. Arguedas A. (2022). Burden of pneumococcal disease due to serotypes covered by the 13-valent and new higher-valent pneumococcal conjugate vaccines in the United States. Vaccine 40, 4700–4708. 10.1016/J.VACCINE.2022.06.024 35753839

[B47] Jafari M. Moghaddam Pour M. Taghizadeh M. Masoudi S. Bayat Z. (2017). Comparative assessment of humoral immune responses of aluminum hydroxide and oil-emulsion adjuvants in Influenza (H9N2) and Newcastle inactive vaccines to chickens. Artif. Cells, Nanomedicine, Biotechnol. 45, 84–89. 10.3109/21691401.2015.1129626 26757848

[B48] Jedrzejas M. J. Lamani E. Becker R. S. (2001). Characterization of selected strains of pneumococcal surface protein A. J. Biol. Chem. 276, 33121–33128. 10.1074/JBC.M103304200 11413137

[B49] Jefferies J. M. C. Johnston C. H. G. Kirkham L. S. Cowan G. J. M. Ross K. S. Smith A. (2007). Presence of nonhemolytic pneumolysin in serotypes of *Streptococcus pneumoniae* associated with disease outbreaks. J. Infect. Dis. 196, 936–944. 10.1086/520091 17703426

[B50] Kamtchoua T. Bologa M. Hopfer R. Neveu D. Hu B. Sheng X. (2013). Safety and immunogenicity of the pneumococcal pneumolysin derivative PlyD1 in a single-antigen protein vaccine candidate in adults. Vaccine 31, 327–333. 10.1016/J.VACCINE.2012.11.005 23153437

[B51] Khan N. Jan A. T. (2017). Towards identifying protective B-cell epitopes: The PspA story. Front. Microbiol. 8, 742. 10.3389/fmicb.2017.00742 28512452 PMC5411445

[B52] Kim G. B. Wang Z. Liu Y. Y. Stavrou S. Mathias A. Goodwin K. J. (2007). A fold-back single-chain diabody format enhances the bioactivity of an anti-monkey CD3 recombinant diphtheria toxin-based immunotoxin. Protein Eng. Des. Sel. 20, 425–432. 10.1093/PROTEIN/GZM040 17693455

[B53] Knittel P. S. Long P. F. Brammall L. Marques A. C. Almeida M. T. Padilla G. (2016). Characterising the enzymatic profile of crude tentacle extracts from the south atlantic jellyfish olindias sambaquiensis (Cnidaria: Hydrozoa). Toxicon 119, 1–7. 10.1016/J.TOXICON.2016.04.048 27169682

[B54] Korz D. J. Rinas U. Hellmuth K. Sanders E. A. Deckwer W.-D. (1995). Simple fed-batch technique for high cell density cultivation of *Escherichia coli* . J. Biotechnol. 39, 59–65. 10.1016/0168-1656(94)00143-Z 7766011

[B55] Laskowski R. A. MacArthur M. W. Moss D. S. Thornton J. M. Iucr (1993). Procheck: A program to check the stereochemical quality of protein structures, 283–291. *urn:issn:0021-8898* 26. 10.1107/S0021889892009944

[B56] Lawrence S. L. Feil S. C. Morton C. J. Farrand A. J. Mulhern T. D. Gorman M. A. (2015). Crystal structure of *Streptococcus pneumoniae* pneumolysin provides key insights into early steps of pore formation. Sci. Rep. 51 (5), 14352–14413. 10.1038/srep14352 PMC4585913 26403197

[B57] Leber T. M. Balkwill F. R. (1997). Zymography: A single-step staining method for quantitation of proteolytic activity on substrate gels. Anal. Biochem. 249, 24–28. 10.1006/ABIO.1997.2170 9193704

[B58] Leroux-Roels G. Maes C. De Boever F. Traskine M. Rüggeberg J. U. Borys D. (2014). Safety, reactogenicity and immunogenicity of a novel pneumococcal protein-based vaccine in adults: A phase I/II randomized clinical study. Vaccine 32, 6838–6846. 10.1016/J.VACCINE.2014.02.052 24607003

[B59] Liang J. G. Su D. Song T.-Z. Zeng Y. Huang W. Wu J. (2020). S-Trimer, a COVID-19 subunit vaccine candidate, induces protective immunity in nonhuman primates. bioRxiv 24, 311027. 10.1101/2020.09.24.311027 PMC7921634 33649323

[B60] Lu P. Feng M. G. (2008). Bifunctional enhancement of a β-glucanase-xylanase fusion enzyme by optimization of peptide linkers. Appl. Microbiol. Biotechnol. 79, 579–587. 10.1007/s00253-008-1468-4 18415095

[B61] Maeda Y. Ueda H. Hara T. Kazami J. Kawano G. Suzuki E. (1996). Expression of a bifunctional chimeric protein A-Vargula hilgendorfii luciferase in mammalian cells. Biotechniques 20, 116–121. 10.2144/96201RR01 8770415

[B62] Maeda Y. Ueda H. Kazami J. Kawano G. Suzuki E. Nagamune T. (1997). Engineering of functional chimeric protein G–VargulaLuciferase. Anal. Biochem. 249, 147–152. 10.1006/ABIO.1997.2181 9212866

[B63] Mancuso R. I. Miyaji E. N. Silva C. C. F. Portaro F. V. Soares-Schanoski A. Ribeiro O. G. (2018). Impaired expression of CXCL5 and matrix metalloproteinases in the lungs of mice with high susceptibility to *Streptococcus pneumoniae* infection. Immun. Inflamm. Dis. 6, 128–142. 10.1002/IID3.205 29119707 PMC5818448

[B64] Marriott H. Mitchell T. Dockrell D. (2008). Pneumolysin: A double-edged sword during the host-pathogen interaction. Curr. Mol. Med. 8, 497–509. 10.2174/156652408785747924 18781957

[B65] Marshall J. E. Faraj B. H. A. Gingras A. R. Lonnen R. Sheikh M. A. El-Mezgueldi M. (2015). The crystal structure of pneumolysin at 2.0 Å resolution reveals the molecular packing of the pre-pore complex. Sci. Rep. 51 (5), 13293–13311. 10.1038/srep13293 PMC4558608 26333773

[B66] Mcchlery S. M. Kerrigan J. Clarke S. C. (2004). Whole gene pneumolysin PCR can be used as a diagnostic assay but cannot predict serotype. Br. J. Biomed. Sci. 61, 31–33. 10.1080/09674845.2004.11978049 15058741

[B67] Minshawi F. Lanvermann S. McKenzie E. Jeffery R. Couper K. Papoutsopoulou S. (2020). The generation of an engineered interleukin-10 protein with improved stability and biological function. Front. Immunol. 11, 1794. 10.3389/fimmu.2020.01794 32849644 PMC7431522

[B68] Mirdita M. Schütze K. Moriwaki Y. Heo L. Ovchinnikov S. Steinegger M. (2022). ColabFold: Making protein folding accessible to all. Nat. Methods 196 19, 679–682. 10.1038/s41592-022-01488-1 PMC9184281 35637307

[B69] Mitsi E. Reiné J. Urban B. C. Solórzano C. Nikolaou E. Hyder-Wright A. D. (2022). *Streptococcus pneumoniae* colonization associates with impaired adaptive immune responses against SARS-CoV-2. J. Clin. Invest. 132, e157124. 10.1172/JCI157124 35139037 PMC8970672

[B70] Miyaji E. N. Oliveira M. L. S. Carvalho E. Ho P. L. (2013). Serotype-independent pneumococcal vaccines. Cell. Mol. Life Sci. 70, 3303–3326. 10.1007/s00018-012-1234-8 23269437 PMC11113425

[B71] Moore M. R. Link-Gelles R. Schaffner W. Lynfield R. Lexau C. Bennett N. M. (2015). Effect of use of 13-valent pneumococcal conjugate vaccine in children on invasive pneumococcal disease in children and adults in the USA: Analysis of multisite, population-based surveillance. Lancet. Infect. Dis. 15, 301–309. 10.1016/S1473-3099(14)71081-3 25656600 PMC4876855

[B72] Moreno A. T. Oliveira M. L. S. Ferreira D. M. Ho P. L. Darrieux M. Leite L. C. C. (2010). Immunization of mice with single PspA fragments induces antibodies capable of mediating complement deposition on different pneumococcal strains and cross-protection. Clin. Vaccine Immunol. 17, 439–446. 10.1128/CVI.00430-09 20089795 PMC2837969

[B73] Nabors G. S. Braun P. A. Herrmann D. J. Heise M. L. Pyle D. J. Gravenstein S. (2000). Immunization of healthy adults with a single recombinant pneumococcal surface protein A (PspA) variant stimulates broadly cross-reactive antibodies to heterologous PspA molecules. Vaccine 18, 1743–1754. 10.1016/S0264-410X(99)00530-7 10699322

[B74] Ogunniyi A. D. Folland R. L. Briles D. E. Hollingshead S. K. Paton J. C. (2000). Immunization of mice with combinations of pneumococcal virulence proteins elicits enhanced protection against challenge with *Streptococcus pneumoniae* . Infect. Immun. 68, 3028–3033. 10.1128/IAI.68.5.3028-3033.2000 10769009 PMC97524

[B75] Ogunniyi A. D. Grabowicz M. Briles D. E. Cook J. Paton J. C. (2007). Development of a vaccine against invasive pneumococcal disease based on combinations of virulence proteins of *Streptococcus pneumoniae* . Infect. Immun. 75, 350–357. 10.1128/IAI.01103-06 17088353 PMC1828427

[B76] Oligbu G. Fry N. K. Ladhani S. N. (2019). The epidemiology and biostatistics of pneumococcus. New York, NY: Humana Press, 215–224. 10.1007/978-1-4939-9199-0_18 30929218

[B77] Oliveira G. S. Oliveira M. L. S. Miyaji E. N. Rodrigues T. C. (2021). Pneumococcal vaccines: Past findings, present work, and future strategies. Vaccines 9. 10.3390/VACCINES9111338 PMC8620834 34835269

[B78] Oliveira M. L. S. Miyaji E. N. Ferreira D. M. Moreno A. T. Ferreira P. C. D. Lima F. A. (2010). Combination of pneumococcal surface protein A (PspA) with whole cell pertussis vaccine increases protection against pneumococcal challenge in mice. PLoS One 5, e10863. 10.1371/journal.pone.0010863 20523738 PMC2877721

[B79] Park C. Kwon E. Y. Choi S. M. Cho S. Y. Byun J. H. Park J. Y. (2017). Comparative evaluation of a newly developed 13-valent pneumococcal conjugate vaccine in a mouse model. Hum. Vaccines Immunother. 13, 1169–1176. 10.1080/21645515.2016.1261772 PMC5443391 27960627

[B80] Peyrani P. Mandell L. Torres A. Tillotson G. S. (2019). The burden of community-acquired bacterial pneumonia in the era of antibiotic resistance. Expert Rev. Respir. Med. 13, 139–152. 10.1080/17476348.2019.1562339 30596308

[B81] Pichichero M. E. (2017). Pneumococcal whole-cell and protein-based vaccines: Changing the paradigm. Expert Rev. Vaccines 16, 1181–1190. 10.1080/14760584.2017.1393335 29130395 PMC6277969

[B82] Platt H. Omole T. Cardona J. Fraser N. J. Mularski R. A. Andrews C. (2022). Safety, tolerability, and immunogenicity of a 21-valent pneumococcal conjugate vaccine, V116, in healthy adults: Phase 1/2, randomised, double-blind, active comparator-controlled, multicentre, US-based trial. Lancet. Infect. Dis. 10.1016/S1473-3099(22)00526-6 36116461

[B83] Pollet J. Chen W. H. Strych U. (2021). Recombinant protein vaccines, a proven approach against coronavirus pandemics. Adv. Drug Deliv. Rev. 170, 71–82. 10.1016/J.ADDR.2021.01.001 33421475 PMC7788321

[B84] Prymula R. Pazdiora P. Traskine M. Rüggeberg J. U. Borys D. (2014). Safety and immunogenicity of an investigational vaccine containing two common pneumococcal proteins in toddlers: A phase II randomized clinical trial. Vaccine 32, 3025–3034. 10.1016/J.VACCINE.2014.03.066 24699466

[B85] Reikofski J. Tao B. Y. (1992). Polymerase chain reaction (PCR) techniques for site-directed mutagenesis. Biotechnol. Adv. 10, 535–547. 10.1016/0734-9750(92)91451-J 14543704

[B86] Ren B. Li J. Genschmer K. Hollingshead S. K. Briles D. E. (2012). The absence of PspA or presence of antibody to PspA facilitates the complement-dependent phagocytosis of pneumococci *in vitro* . Clin. Vaccine Immunol. 19, 1574–1582. 10.1128/CVI.00393-12 22855389 PMC3485889

[B87] Rodrigo C. Bewick T. Sheppard C. Greenwood S. Mckeever T. M. Trotter C. L. (2015). Impact of infant 13-valent pneumococcal conjugate vaccine on serotypes in adult pneumonia. Eur. Respir. J. 45, 1632–1641. 10.1183/09031936.00183614 25792633

[B88] Rodrigues T. C. Oliveira M. L. S. Soares-Schanoski A. Chavez-Rico S. L. Figueiredo D. B. Gonçalves V. M. (2018). Mucosal immunization with PspA (Pneumococcal surface protein A)-adsorbed nanoparticles targeting the lungs for protection against pneumococcal infection. PLoS One 13, e0191692. 10.1371/JOURNAL.PONE.0191692 29360883 PMC5779684

[B89] Roth G. A. Abate D. Abate K. H. Abay S. M. Abbafati C. Abbasi N. (2018). Global, regional, and national age-sex-specific mortality for 282 causes of death in 195 countries and territories, 1980–2017: A systematic analysis for the global burden of disease study 2017. Lancet 392, 1736–1788. 10.1016/S0140-6736(18)32203-7 30496103 PMC6227606

[B90] Seeger A. Schneppe B. McCarthy J. E. G. Deckwer W. D. Rinas U. (1995). Comparison of temperature- and isopropyl-β-d-thiogalacto-pyranoside-induced synthesis of basic fibroblast growth factor in high-cell-density cultures of recombinant *Escherichia coli* . Enzyme Microb. Technol. 17, 947–953. 10.1016/0141-0229(94)00123-9

[B91] Senkovich O. Cook W. J. Mirza S. Hollingshead S. K. Protasevich I. I. Briles D. E. (2007). Structure of a complex of human lactoferrin N-lobe with pneumococcal surface protein a provides insight into microbial defense mechanism. J. Mol. Biol. 370, 701–713. 10.1016/j.jmb.2007.04.075 17543335 PMC5356469

[B92] Sippl M. J. (1993). Recognition of errors in three-dimensional structures of proteins. Proteins Struct. Funct. Bioinforma. 17, 355–362. 10.1002/PROT.340170404 8108378

[B93] Sreerama N. Woody R. W. (2000). Estimation of protein secondary structure from circular dichroism spectra: Comparison of CONTIN, SELCON, and CDSSTR methods with an expanded reference set. Anal. Biochem. 287, 252–260. 10.1006/abio.2000.4880 11112271

[B94] Troeger C. Forouzanfar M. Rao P. C. Khalil I. Brown A. Swartz S. (2017). Estimates of the global, regional, and national morbidity, mortality, and aetiologies of lower respiratory tract infections in 195 countries: A systematic analysis for the global burden of disease study 2015. Lancet. Infect. Dis. 17, 1133–1161. 10.1016/S1473-3099(17)30396-1 28843578 PMC5666185

[B95] Vandooren J. Geurts N. Martens E. Van Den Steen P. E. Opdenakker G. (2013). Zymography methods for visualizing hydrolytic enzymes. Nat. Methods 103 (10), 211–220. 10.1038/nmeth.2371 23443633

[B96] Wahl B. O’Brien K. L. Greenbaum A. Majumder A. Liu L. Chu Y. (2018). Burden of *Streptococcus pneumoniae* and Haemophilus influenzae type b disease in children in the era of conjugate vaccines: Global, regional, and national estimates for 2000–15. Lancet Glob. heal. 6, e744–e757. 10.1016/S2214-109X(18)30247-X PMC6005122 29903376

[B97] Waight P. A. Andrews N. J. Ladhani S. N. Sheppard C. L. Slack M. P. E. Miller E. (2015). Effect of the 13-valent pneumococcal conjugate vaccine on invasive pneumococcal disease in england and wales 4 years after its introduction: An observational cohort study. Lancet. Infect. Dis. 15, 535–543. 10.1016/S1473-3099(15)70044-7 25801458

[B98] Whitmore L. Wallace B. A. (2004). DICHROWEB, an online server for protein secondary structure analyses from circular dichroism spectroscopic data. Nucleic Acids Res. 32, W668–W673. 10.1093/nar/gkh371 15215473 PMC441509

[B99] Wiederstein M. Sippl M. J. (2007). ProSA-web: Interactive web service for the recognition of errors in three-dimensional structures of proteins. Nucleic Acids Res. 35, W407–W410. 10.1093/NAR/GKM290 17517781 PMC1933241

[B100] World Health Organization (2019). Pneumonia. Available at: https://www.who.int/news-room/fact-sheets/detail/pneumonia (Accessed November 5, 2022).

[B101] Yother J. Handsome G. L. Briles D. E. (1992). Truncated forms of PspA that are secreted from *Streptococcus pneumoniae* and their use in functional studies and cloning of the pspA gene. J. Bacteriol. 174, 610–618. 10.1128/jb.174.2.610-618.1992 1729250 PMC205756

[B102] Yu K. Liu C. Kim B. G. Lee D. Y. (2015). Synthetic fusion protein design and applications. Biotechnol. Adv. 33, 155–164. 10.1016/J.BIOTECHADV.2014.11.005 25450191

[B103] Zhao H. L. Yao X. Q. Xue C. Wang Y. Xiong X. H. Liu Z. M. (2008). Increasing the homogeneity, stability and activity of human serum albumin and interferon-α2b fusion protein by linker engineering. Protein Expr. Purif. 61, 73–77. 10.1016/J.PEP.2008.04.013 18541441

