# Supplementary material for: Peptide linker increased the stability of pneumococcal fusion protein vaccine candidate
Source: Front Bioeng Biotechnol. 2023 Jan 26;11:1108300. doi: 10.3389/fbioe.2023.1108300 (PMC9909212; doi:10.3389/fbioe.2023.1108300)
Supplement: Supplementary file 1 [file DataSheet1.PDF]

Supplementary material

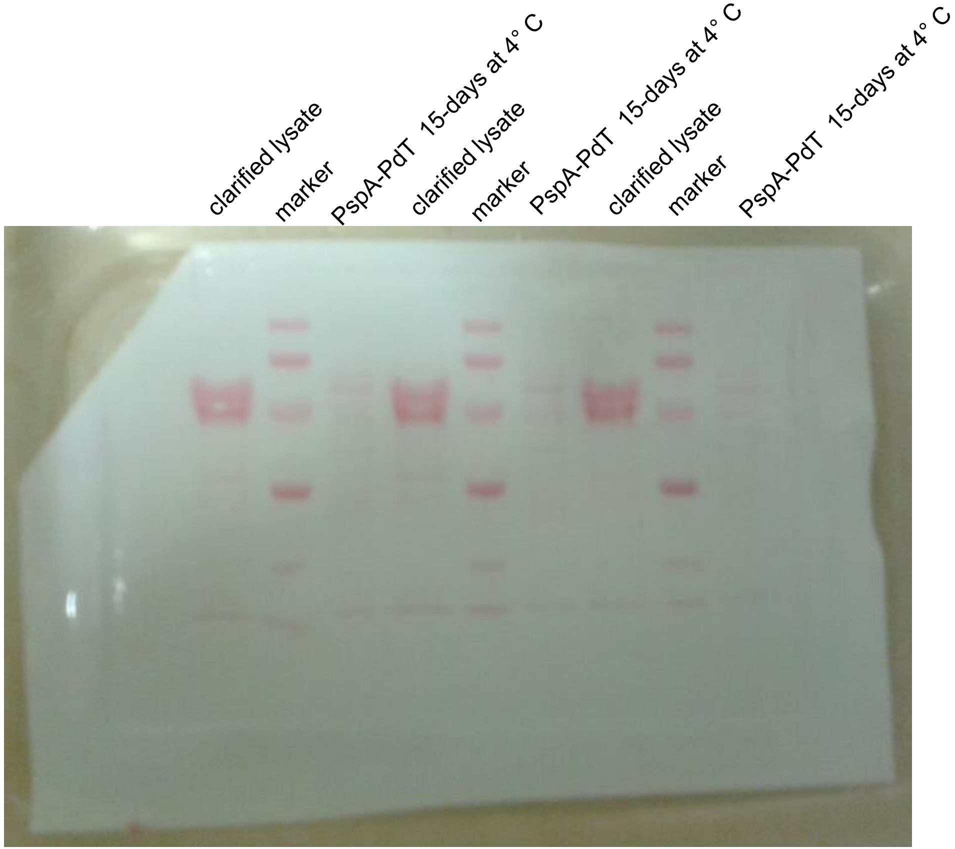

Supplementary Figure 1. Nitrocellulose membrane stained with Ponceau S before performing the western blotting to verify PspA-PdT stability using different antibodies for detection. Clarified lysate, marker and 15-days purified PspA-PdT stored at 4° C were loaded in triplicates, transferred, the membrane was split in three parts for detection with anti-PspA94, anti-PWCV (pneumococcal whole cell vaccine) and anti-PdT.

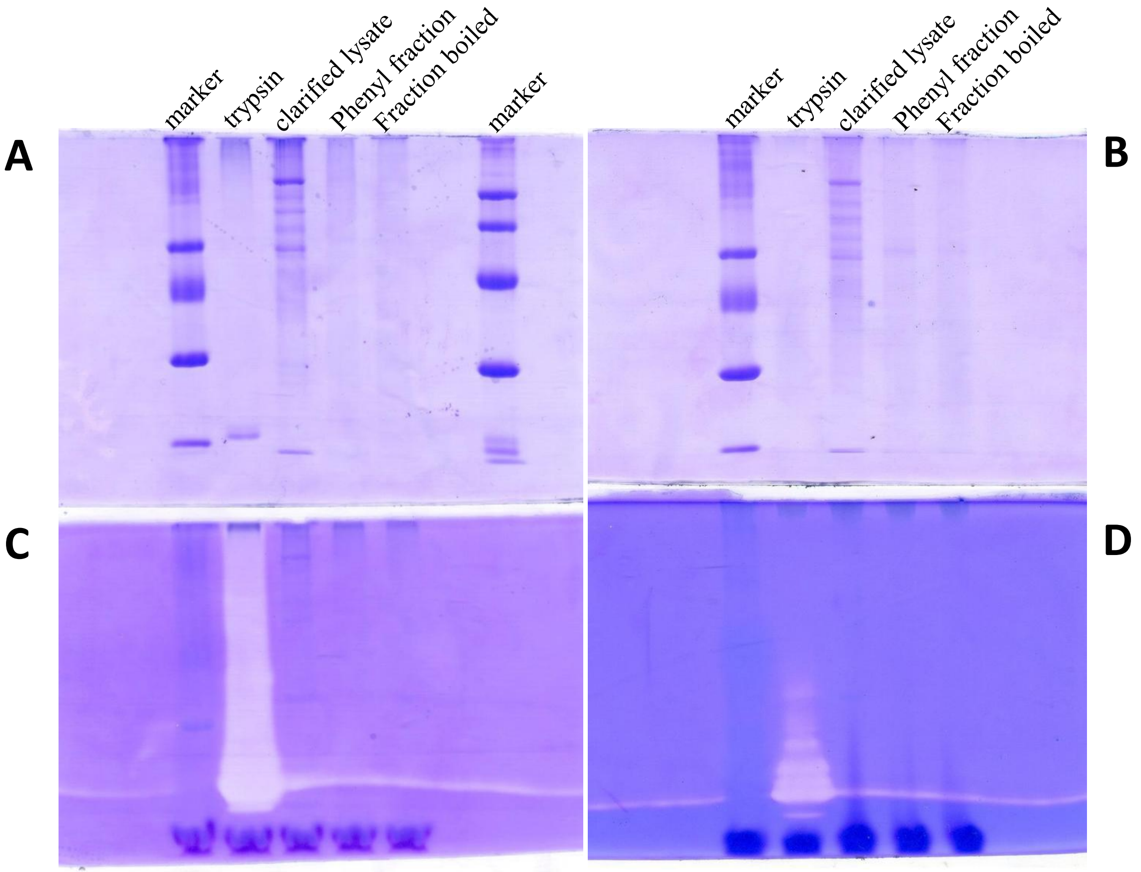

Supplementary Figure 2. Protease activity detection assays. **(A and B)** SDS-PAGE (10%) control gels without substrates with Coomassie staining, **(C)** in-gel zymography with 0.1% gelatin as substrate, and **(D)** in-gel zymography with 0.1% casein as substrate. Samples applied to the gels are indicated on the top: molecular weight marker; trypsin as positive control; recently clarified lysate; partially purified sample from a Phenyl-Sepharose elution fraction where degradation was observed; the same Phenyl-Sepharose fraction boiled for 10 min before load.

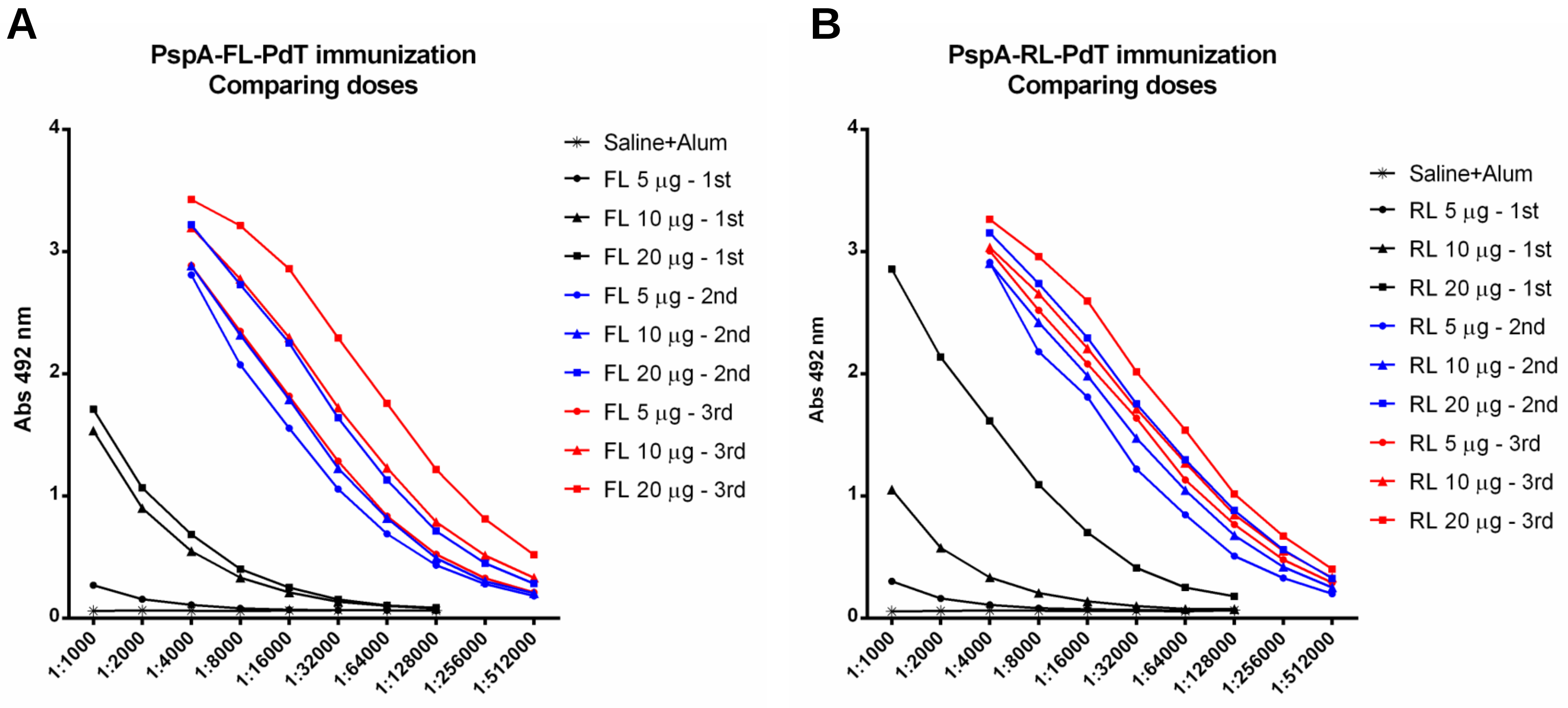

Supplementary Figure 3. Titration curves of specific IgG obtained by ELISA carried out from pooled sera from each group of mice immunized subcutaneously with three doses of 5 µg, 10 µg or 20 µg of **(A)** rPspA-FL-PdT or **(B)** rPspA-RL-PdT. The animals were immunized with 15-days interval with the indicated amount of fusion protein or control containing only the adjuvant Alum (50 µg) in saline. Blood was collected 14 days after each immunization. Plates were coated with either rPspA-FL-PdT or rPspA-RL-PdT, accordingly with the groups analyzed.

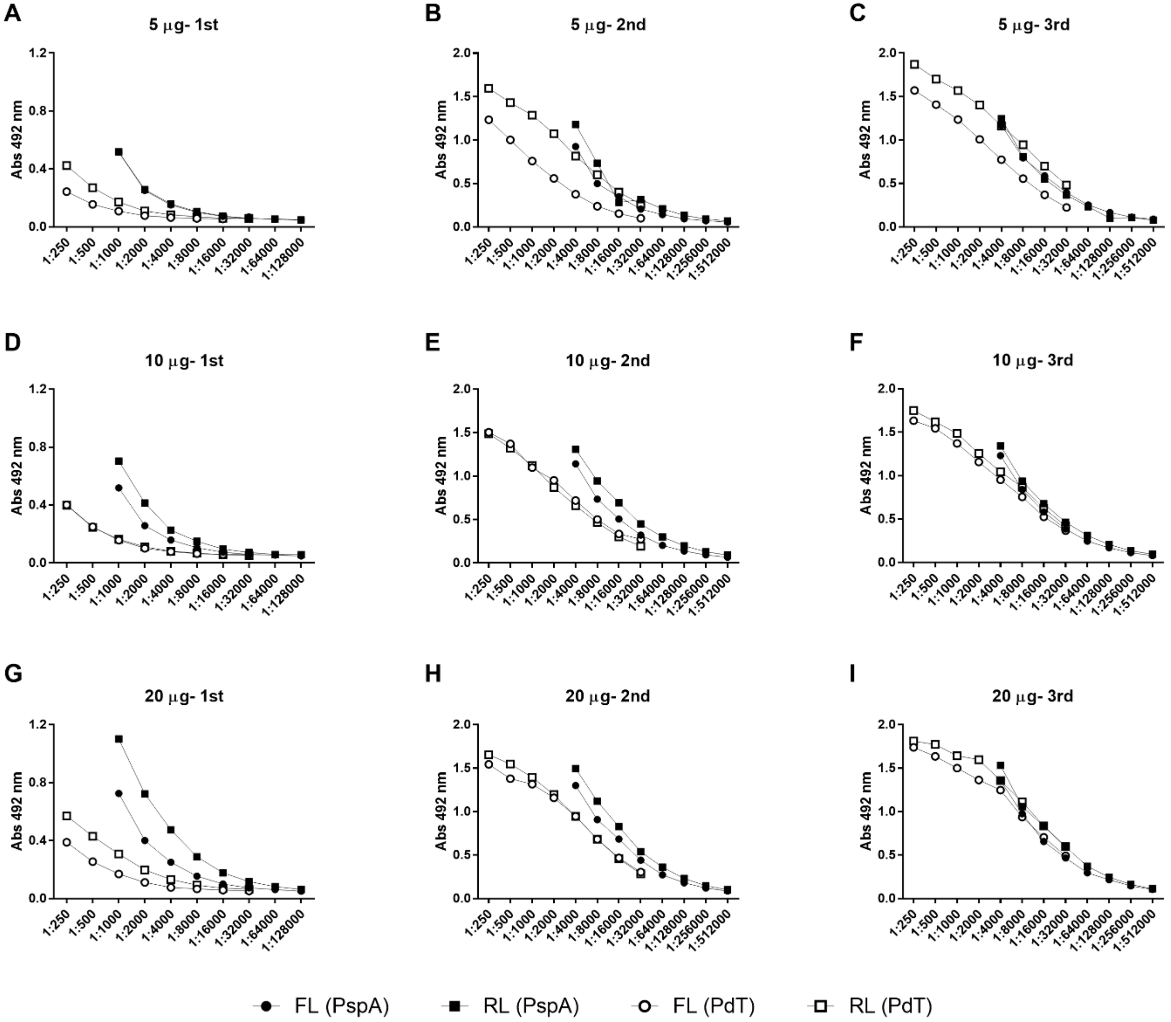

Supplementary Figure 4. Titration curves of specific IgG obtained by ELISA carried out from pooled sera from each group of mice immunized subcutaneously with three doses of 5 µg, 10 µg or 20 µg of rPspA-FL-PdT or rPspA-RL-PdT. The animals were immunized with 15-days interval with the indicated amount of fusion protein or control containing only the adjuvant Alum (50 µg) in saline. Blood was collected 14 days after each immunization. Plates were coated with either PspA94 or PdT, and all the groups were analyzed with each protein. **(A, B and C)** First, second and third doses of groups immunized with 5 µg of fusion proteins, respectively. **(D, E and F)** First, second and third doses of groups immunized with 10 µg of fusion proteins, respectively. **(G, H and I)** First, second and third doses of groups immunized with 20 µg of fusion proteins, respectively.

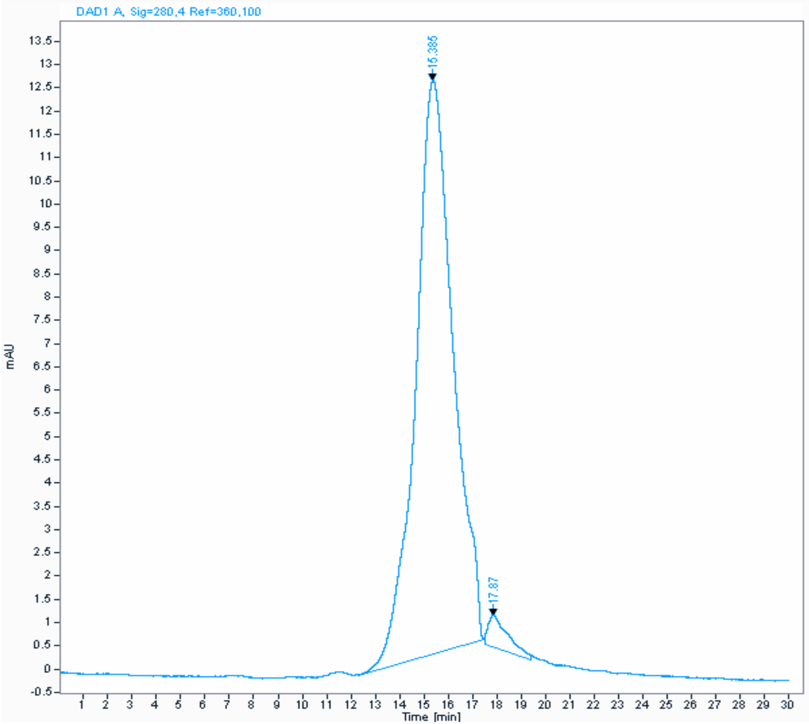

RT [min]	Width [min]	Area	Height	Area%
15.385	1.5792	1323.2212	12.3378	97.1055
17.870	0.9514	39.4419	0.6909	2.8945
17,881	Sum	1362.6631	0.6422	1.8402

Supplementary Figure 5. Analysis of rPspA-FL-PdT stored for 18 months at -20 °C by size exclusion high performance liquid chromatography. Chromatogram detected by UV_{280} was integrated and analyzed regarding %area of the peaks. The retention time of 15.385 min corresponds to the fusion protein.

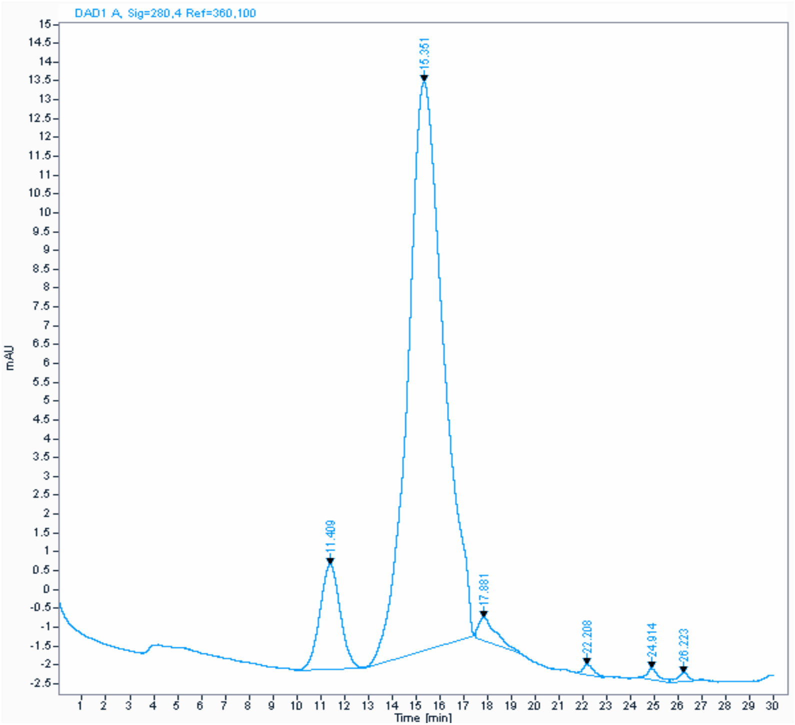

RT [min]	Width [min]	Area	Height	Area%
11,409	0.9065	163.9983	2.7709	9.1598
15,351	1.5332	1568.2186	15.0803	87.5894
17,881	0.8551	32.9468	0.6422	1.8402
22,208	0.5098	8.3296	0.2723	0.4652
24,914	0.5403	9.3631	0.2888	0.5230
26,223	0.5640	7.5645	0.2236	0.4225
	Sum	1790.4210		

Supplementary Figure 6. Analysis of rPspA-FL-PdT stored for 18 months at 4 °C by size exclusion high performance liquid chromatography. Chromatogram detected by UV_{280} was integrated and analyzed regarding %area of the peaks. The retention time of 15.351 min corresponds to the fusion protein.

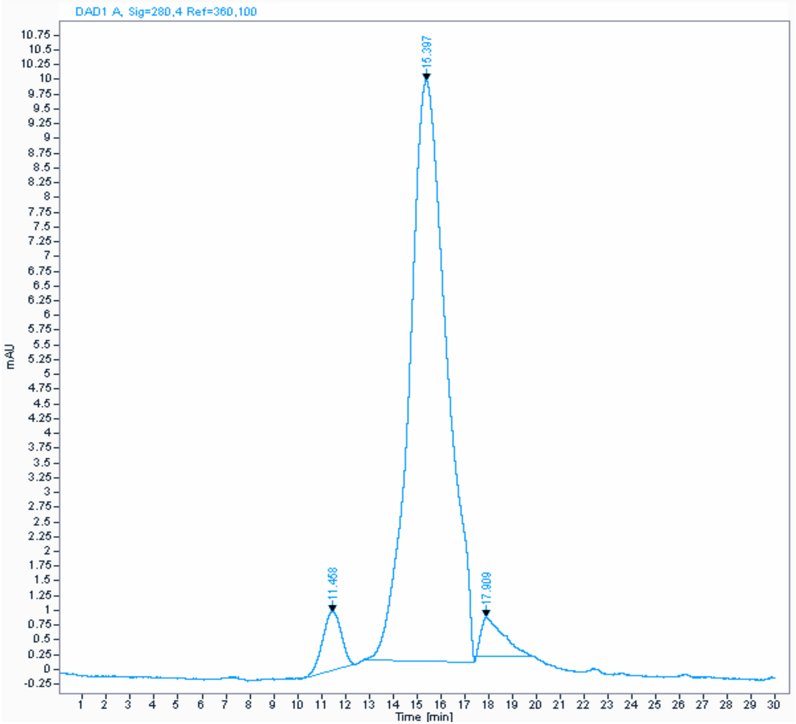

RT [min]	Width [min]	Area	Height	Area%
11.458	0.8898	53.3813	0.9999	4.5855
15.397	1.8066	1065.8951	9.8334	91.5605
17.909	1.1018	44.8671	0.6787	3.8541
	Sum	1164.1436		

Supplementary Figure 7. Analysis of rPspA-RL-PdT stored for 18 months at -20 °C by size exclusion high performance liquid chromatography. Chromatogram detected by UV_{280} was integrated and analyzed regarding %area of the peaks. The retention time of 15.397 min corresponds to the fusion protein.

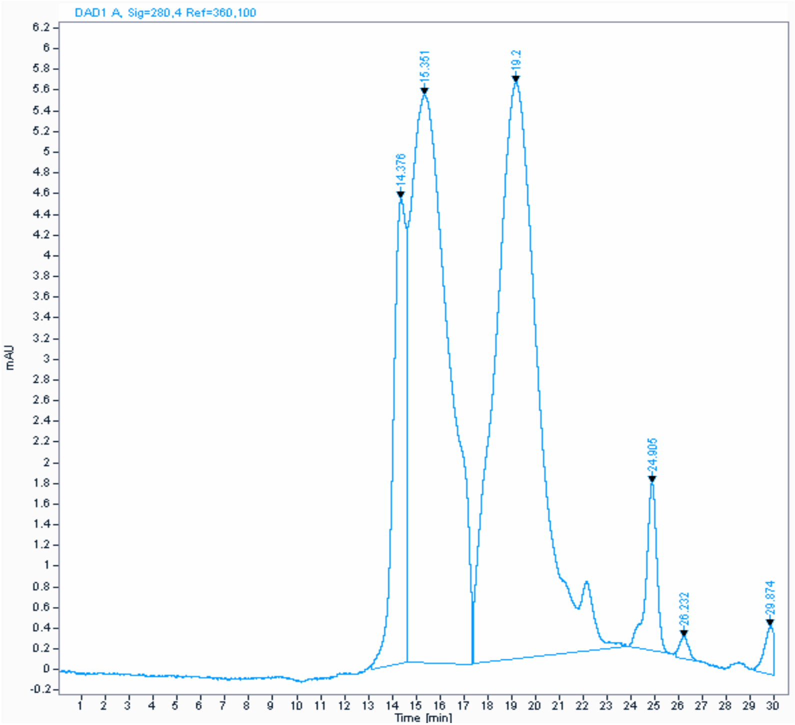

RT [min]	Width [min]	Area	Height	Area%
14.376	0.6382	172.1022	4.4944	11.0679
15.351	1.8239	600.6453	5.4887	38.6276
19.200	1.8896	714.2866	5.5646	45.9358
24.905	0.4750	50.7067	1.6298	3.2610
26.232	0.4375	5.6662	0.2159	0.3644
29.874	0.4152	11.5587	0.4640	0.7433
	Sum	1554.9657		

Supplementary Figure 8. Analysis of rPspA-RL-PdT stored for 18 months at 4 °C by size exclusion high performance liquid chromatography. Chromatogram detected by UV_{280} was integrated and analyzed regarding %area of the peaks. The retention time of 15.351 min corresponds to the fusion protein.

Raw data

Full scan of the entire original gels and blots presented in the article are displayed below

Labels are given only for the lanes employed to elaborate the figures, other lanes were not labeled to keep conciseness

Uncropped gels of Figure 1A

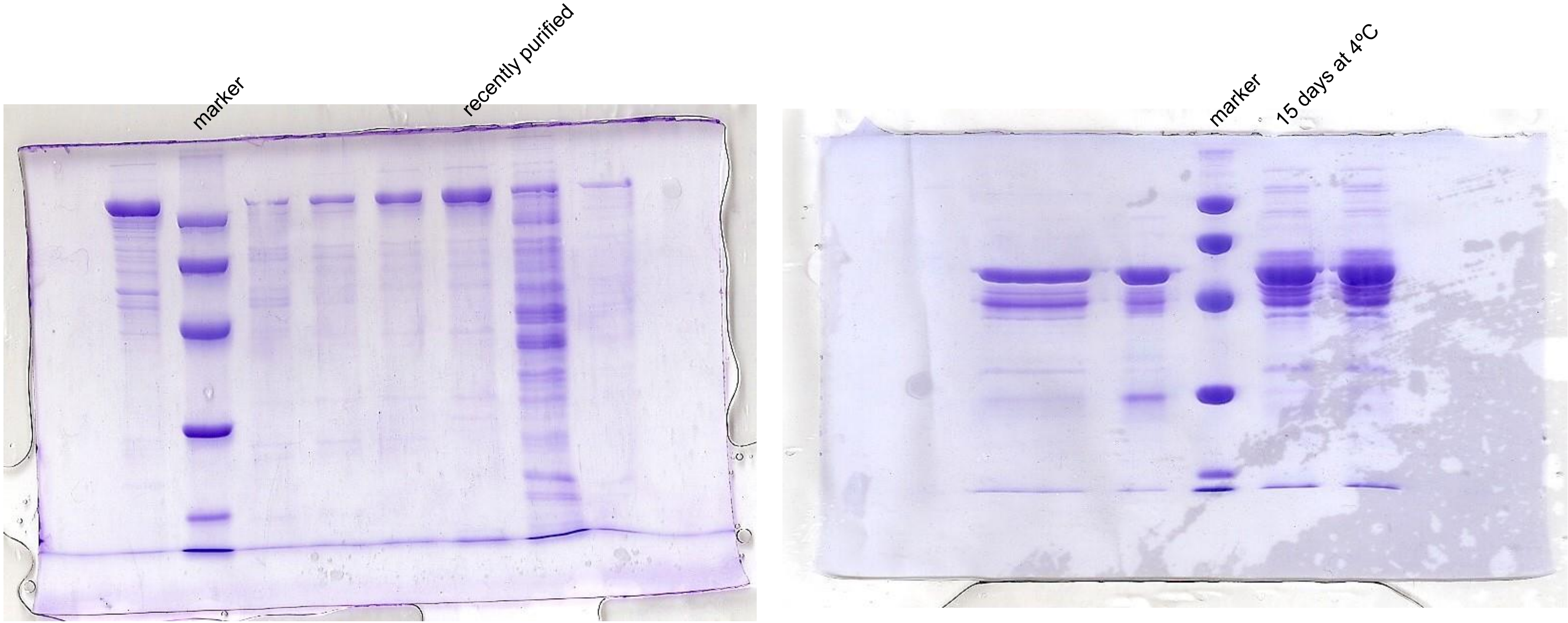

Figure 1. Degradation of PspA-PdT observed during purification trial. **A)** SDS-PAGE 10% assessing PspA-PdT purity and stability. Lanes loaded with PspA-PdT sample after purification by hydrophobic interaction, **recently purified** and 15 days after storage at 4 °C were indicated.

Uncropped gels of Figure 3 B-E

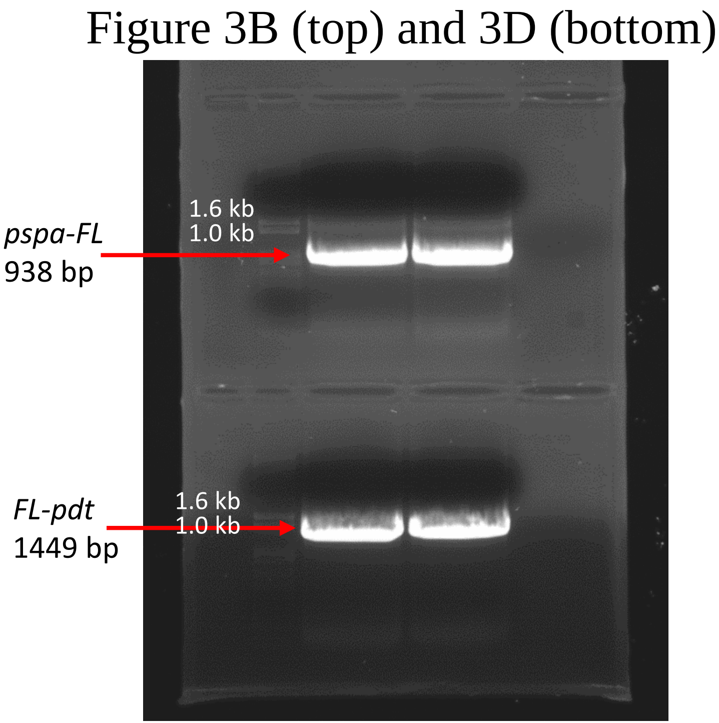

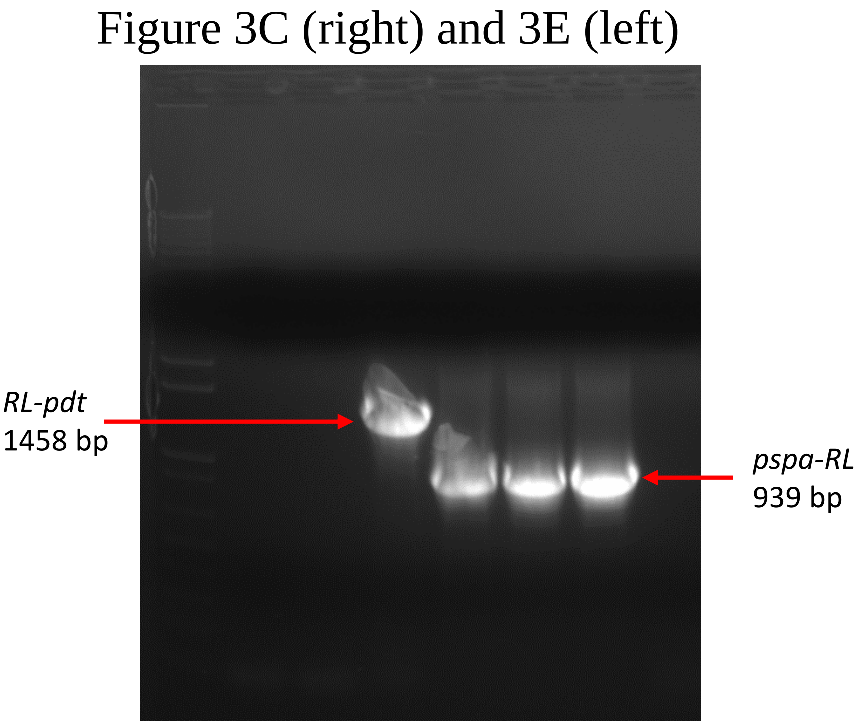

Figure 3. Agarose gels with PCR products. **(B, C)** Fragments of *pspa* amplified with a part of flexible linker (FL) and rigid linker (RL), respectively. **(D, E)** Fragments of *pdt* amplified with a part of FL and RL, respectively.

Uncropped gels of Figure 3F-G

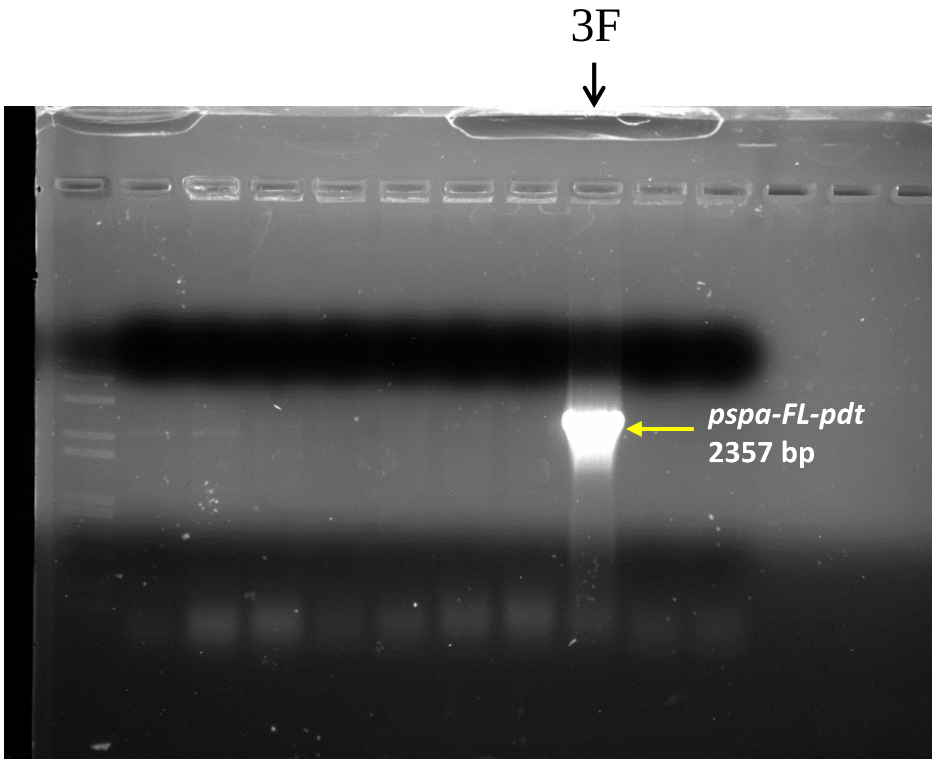

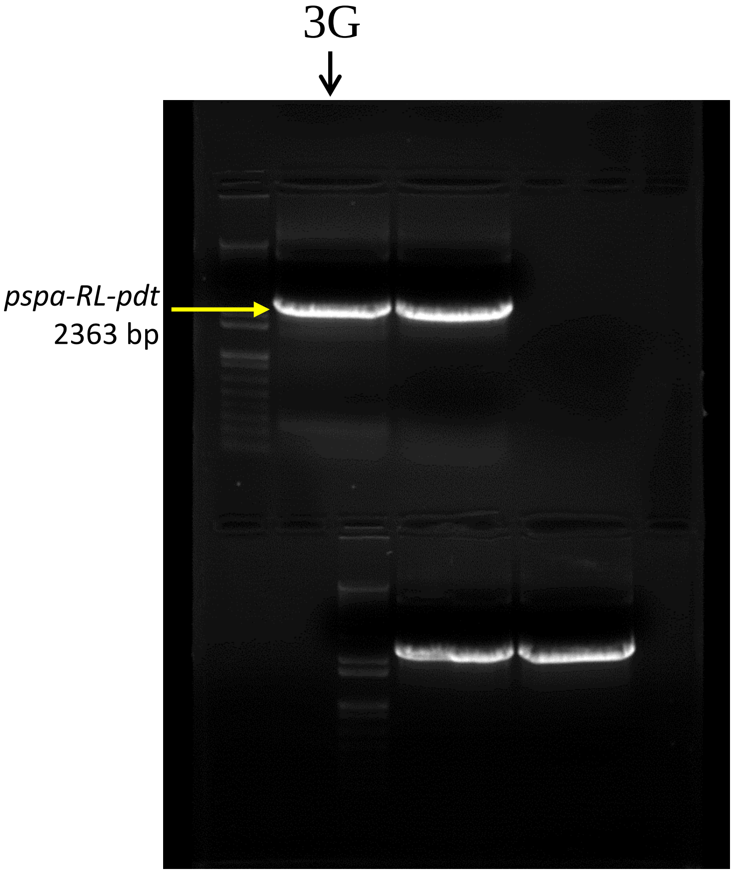

Figure 3. Agarose gels with PCR products. **(F, G)** PCR product after amplification of complete *pspa-FL-pdt* and *pspa-RL-pdt*, respectively.

Uncropped gels of Figure 4A

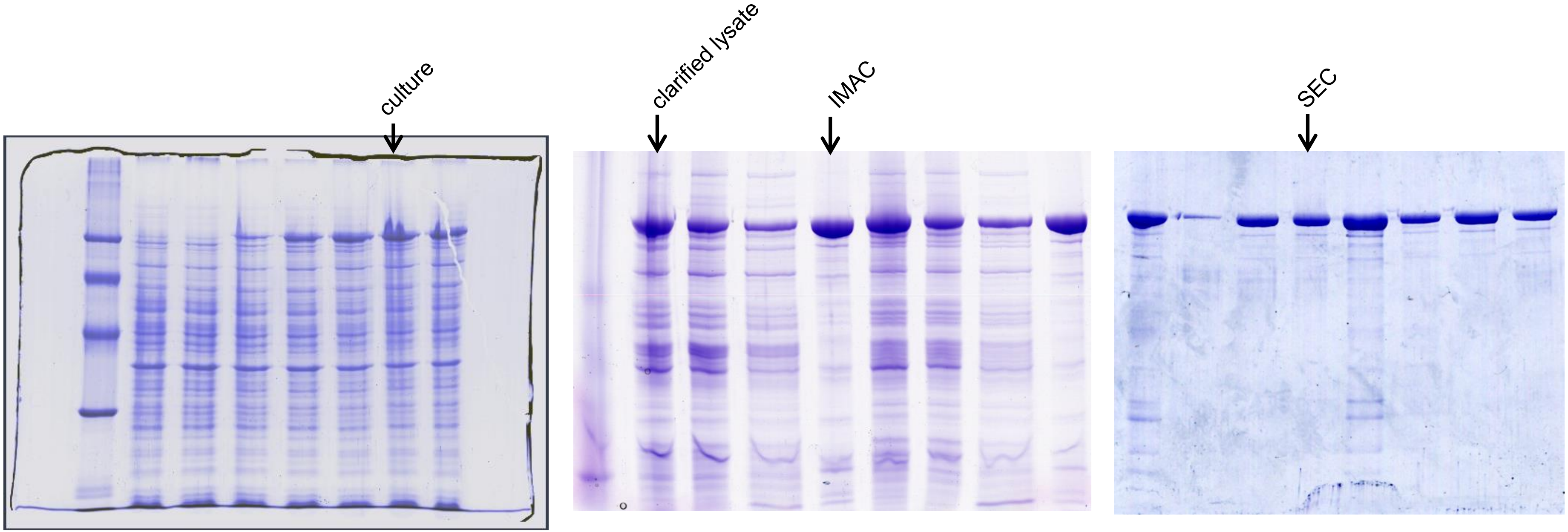

Figure 4. SDS-PAGE (10% gels) for analysis of each purification step for **PspA-FL-PdT**. The arrows show the lanes used to compose Figure **4A**: 1) total protein from induced culture (40% purity); 2) soluble fraction of clarified lysate (59% purity); 3) elution of IMAC (78% purity); 4) elution of SEC (95% purity).

Uncropped gels of Figure 4B

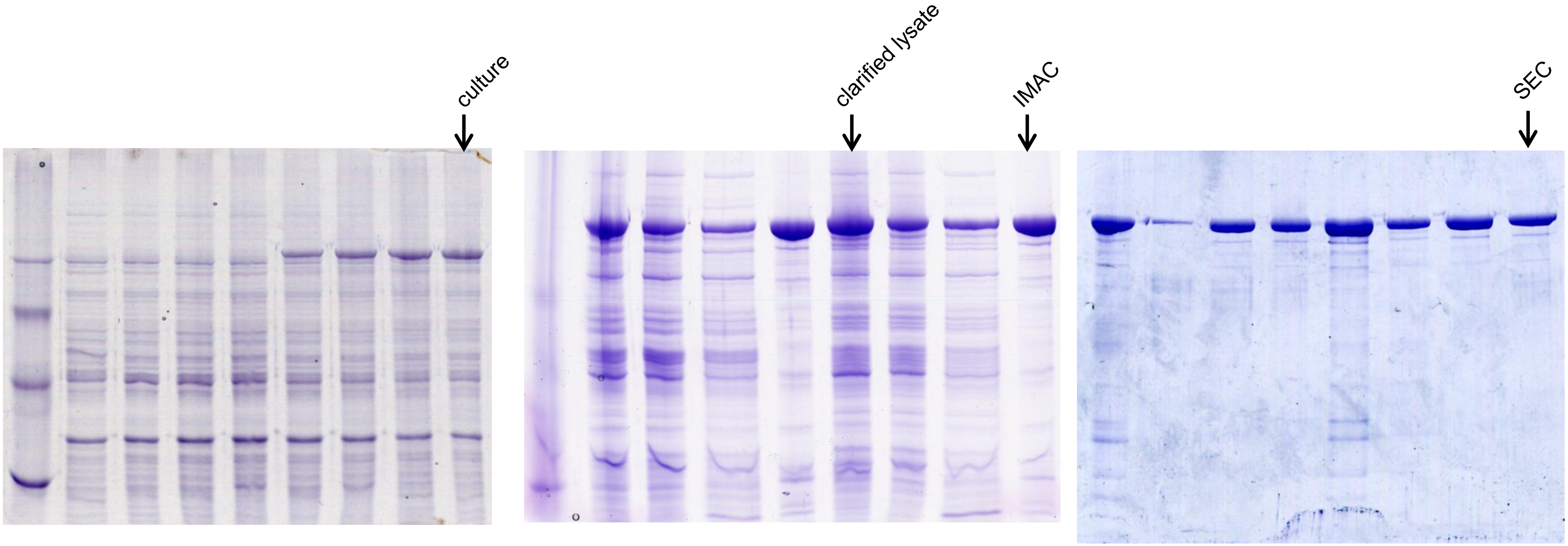

Figure 4. SDS-PAGE (10% gels) for analysis of each purification step of **PspA-RL-PdT**. The arrows show the lanes used to compose Figure **4B**: 1) total protein from induced culture (37% purity); 2) soluble fraction of clarified lysate (57% purity); 3) elution of IMAC (83% purity); 4) elution of SEC (95% purity).

Uncropped gels of Figure 5A

marker

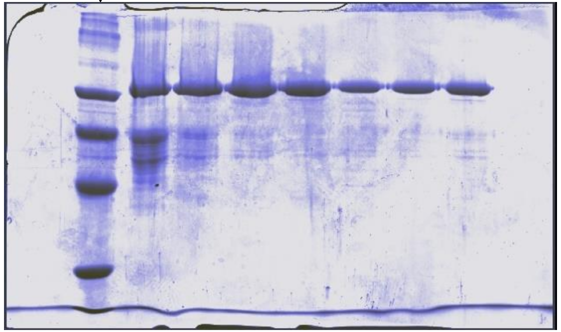

after purification

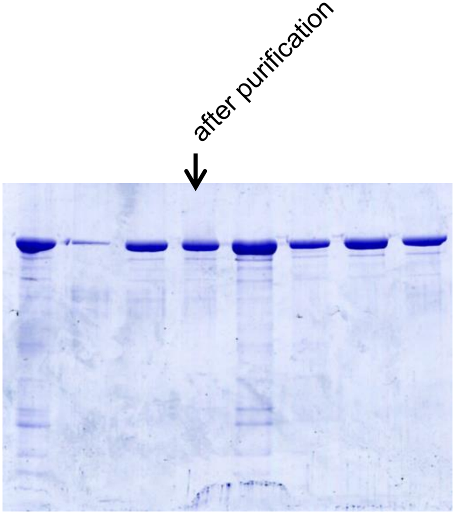

WB 12 months

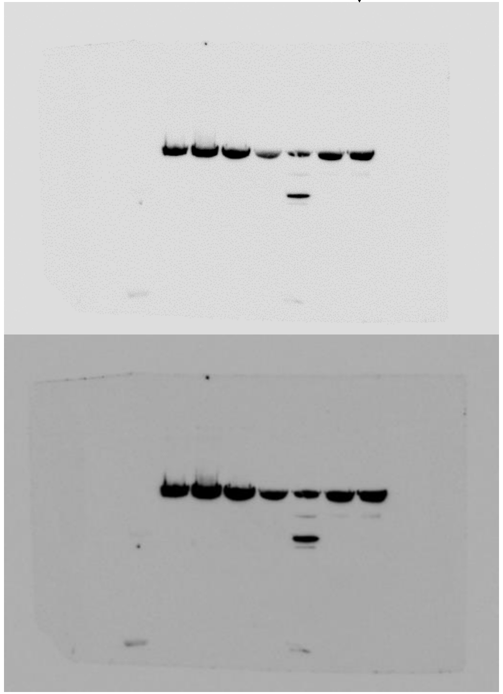

(less bright to see the membrane)

12 months

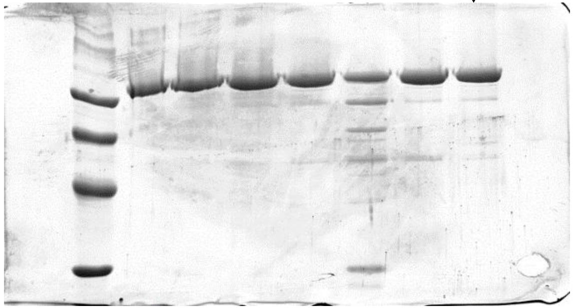

Figure 5. Stability periodic evaluation of the fusion proteins by SDS-PAGE (10% gels) and Western blot of **PspA-FL-PdT stored at 4 °C**. The arrows show the lanes used to compose Figure **5A**: molecular marker; PspA-FL-PdT just after purification (1 day); PspA-FL-PdT 12 months after purification; western blot (WB) of PspA-FL-PdT 12 months after purification.

Uncropped gels of Figure 5B

12 months

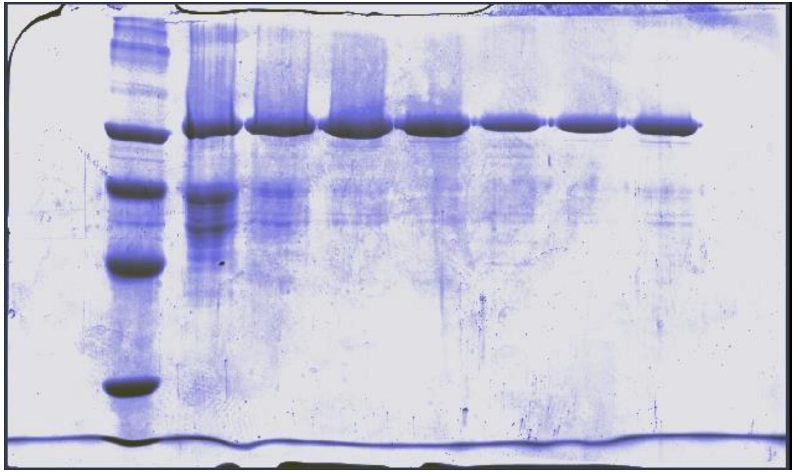

WB 12 months

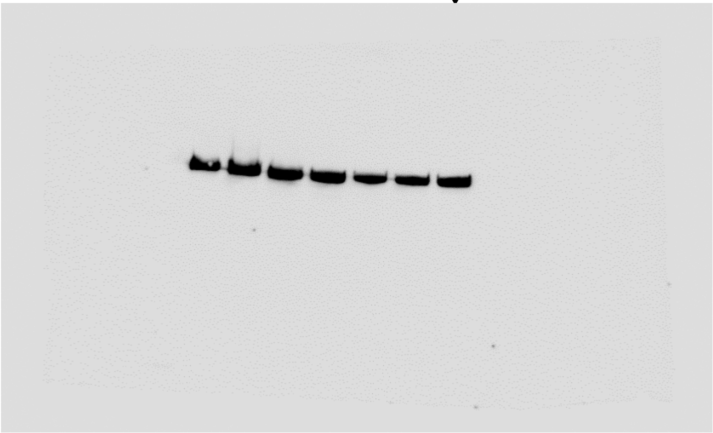

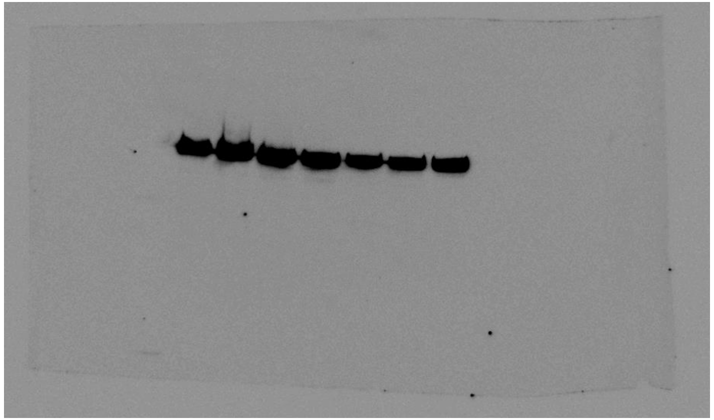

(less bright to see the membrane)

Figure 5. Stability periodic evaluation of the fusion proteins by SDS-PAGE and western blot of **PspA-FL-PdT stored at -20 °C**. The arrows show the lanes used to compose Figure **5B**: PspA-FL-PdT 12 months after purification and western blot (WB) of PspA-FL-PdT 12 months after purification.

Uncropped gels of Figure 5C

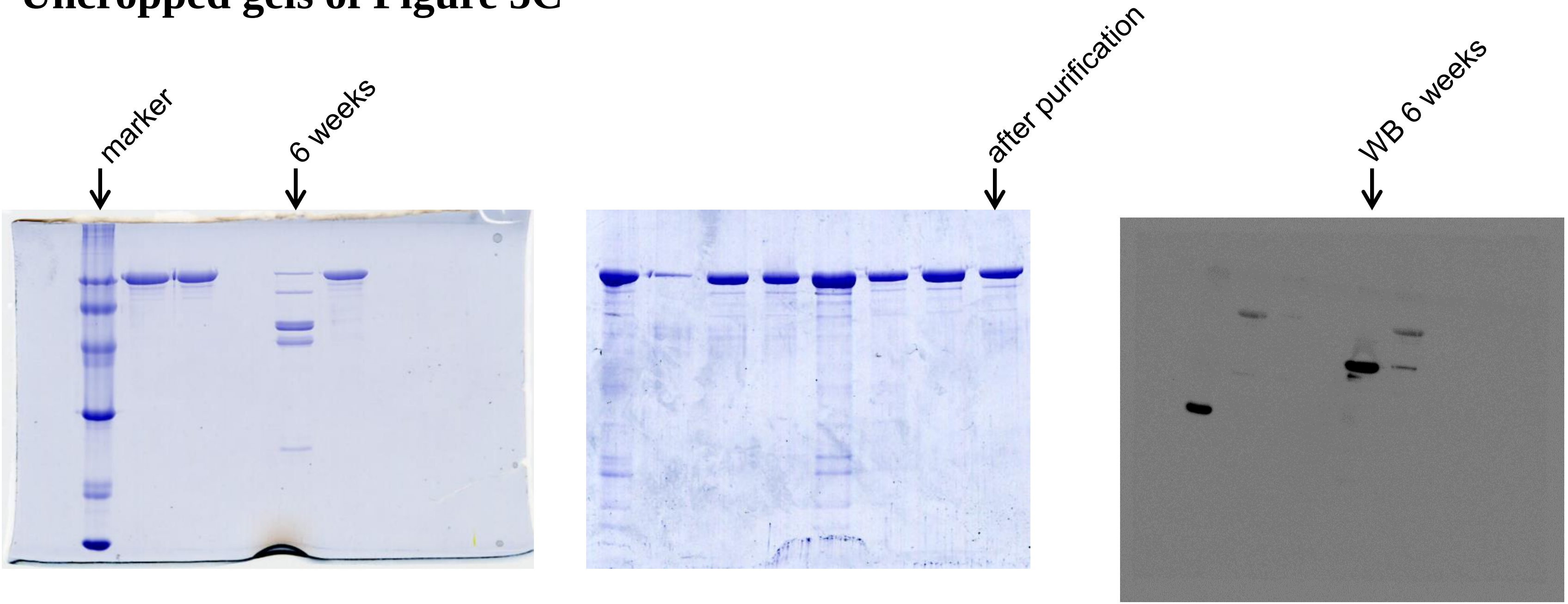

Figure 5. Stability periodic evaluation of the fusion proteins by SDS-PAGE and western blot of **PspA-RL-PdT stored at 4 °C**. The arrows show the lanes used to compose Figure **5C**: molecular marker; PspA-RL-PdT was evaluated just after purification (1 day); PspA-RL-PdT 6 weeks after purification; western blot (WB) of PspA-RL-PdT 6 weeks after purification.

Uncropped gels of Figure 5D

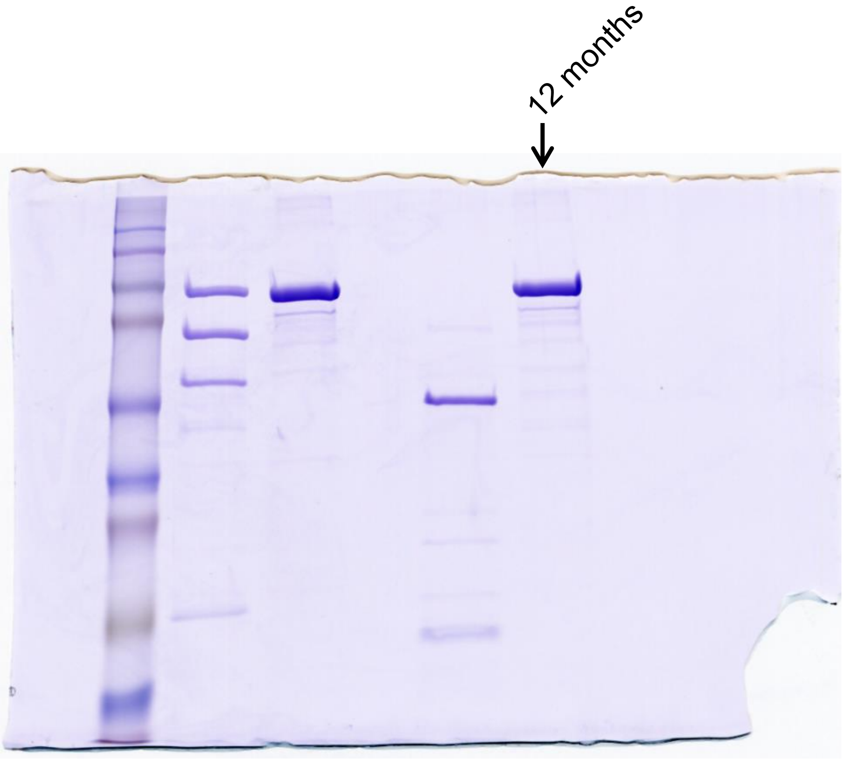

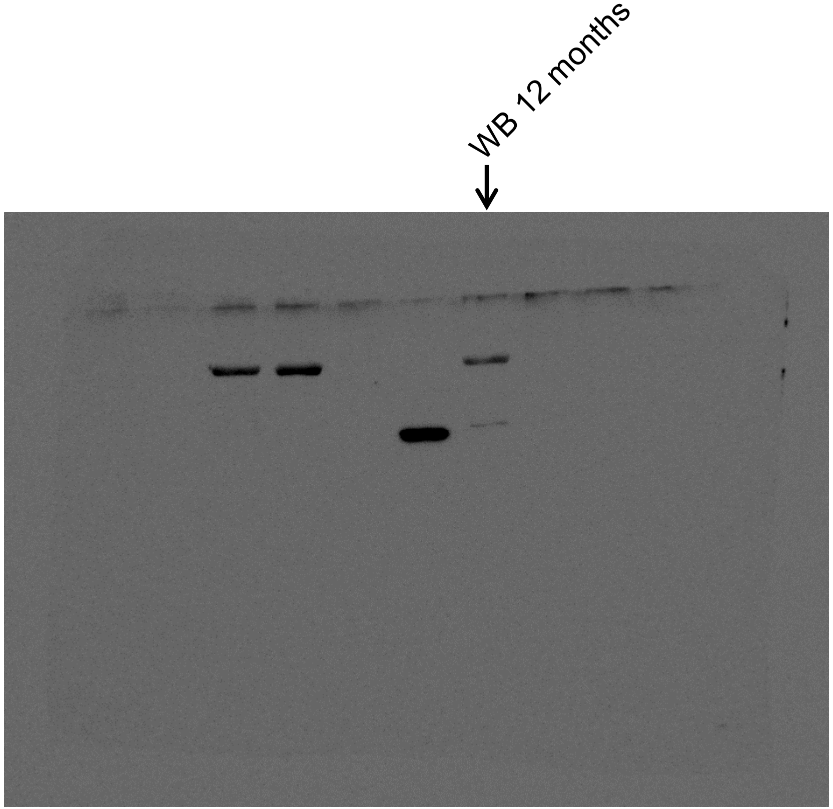

Figure 5. Stability periodic evaluation of the fusion proteins by SDS-PAGE and western blot of **PspA-RL-PdT stored at -20 °C**. The arrows show the lanes used to compose Figure **5D**: PspA-RL-PdT 12 months after purification and western blot (WB) of PspA-RL-PdT 12 months after purification.
